# Association of hypoxia inducible factor 1-Alpha gene polymorphisms with multiple disease risks: A comprehensive meta-analysis

**DOI:** 10.1371/journal.pone.0273042

**Published:** 2022-08-16

**Authors:** Md. Harun-Or-Roshid, Md. Borqat Ali, Jesmin, Md. Nurul Haque Mollah

**Affiliations:** 1 Bioinformatics Laboratory, Department of Statistics, University of Rajshahi, Rajshahi, Bangladesh; 2 Department of Genetic Engineering and Biotechnology, University of Dhaka, Dhaka, Bangladesh; UNITED STATES

## Abstract

*HIF1A* gene polymorphisms have been confirmed the association with cancer risk through the statistical meta-analysis based on single genetic association (SGA) studies. A good number SGA studies also investigated the association of *HIF1A* gene with several other diseases, but no researcher yet performed statistical meta-analysis to confirm this association more accurately. Therefore, in this paper, we performed a statistical meta-analysis to draw a consensus decision about the association of *HIF1A* gene polymorphisms with several diseases except cancers giving the weight on large sample size. This meta-analysis was performed based on 41 SGA study’s findings, where the polymorphisms rs11549465 (1772 C/T) and rs11549467 (1790 G/A) of *HIF1A* gene were analyzed based on 11544 and 7426 cases and 11494 and 7063 control samples, respectively. Our results showed that the 1772 C/T polymorphism is not significantly associated with overall disease risks. The 1790 G/A polymorphism was significantly associated with overall diseases under recessive model (*AA* vs. *AG* + *GG*), which indicates that the *A* allele is responsible for overall diseases though it is recessive. The subgroup analysis based on ethnicity showed the significant association of 1772 C/T polymorphism with overall disease for Caucasian population under the all genetic models, which indicates that the *C* allele controls overall diseases. The ethnicity subgroup showed the significant association of 1790 G/A polymorphism with overall disease for Asian population under the recessive model (*AA* vs. *AG* + *GG*), which indicates that the *A* allele is responsible for overall diseases. The subgroup analysis based on disease types showed that 1772 C/T is significantly associated with chronic obstructive pulmonary disease (COPD) under two genetic models (*C* vs. *T* and *CC* vs. *CT* + *TT*), skin disease under two genetic models (*CC* vs. *TT* and *CC* + *CT* vs. *TT)*, and diabetic complications under three genetic models (*C* vs. *T*, *CT* vs. *TT* and *CC* + *CT* vs. *TT)*, where *C* allele is high risk factor for skin disease and diabetic complications (since, ORs > 1), but low risk factor for COPD (since, ORs < 1). Also the 1790 G/A variant significantly associated with the subgroup of cardiovascular disease (CVD) under homozygote model, diabetic complications under allelic and homozygote models, and other disease under four genetic models, where the *A* is high risk factor for diabetic complications and low risk factor for CVD. Thus, this study provided more evidence that the *HIF1A* gene is significantly associated with COPD, CVD, skin disease and diabetic complications. These might be the severe comorbidities and risk factors for multiple cancers due to the effect of *HIF1A* gene and need further investigations accumulating large number of studies.

## Introduction

In the scientific community, hypoxia-inducible factor 1α (*HIF1A*), a transcription factor, has been a research focus to explain its role in oxygen sensing under normal and hypoxic conditions. Many aspects of Human physiology need to match oxygen supply to cellular metabolism and presumably regulate gene expression by sensing oxygen [1]. *HIF1A* regulates the expression of hundreds of genes [2, 3] involved in many biological processes, including neovascularization, angiogenesis, cytoskeletal structure, apoptosis, adhesion, migration, invasion, metastasis, glycolysis, and metabolic bioenergetics [4–6]. Low oxygen levels or hypoxia represent an important microenvironment condition to affect the pathology of many human diseases, including cancer, diabetes, aging, and stroke/ ischemia [7, 8]. *HIF1A* 1772 C/T (rs11549465) and 1790 G/A (rs11549467) single nucleotide polymorphisms (SNPs) have been identified in association with different types of cancers [9–14]. In recent years, a study also reviewed the association of *HIF1A* 1772 C/T and 1790 G/A polymorphisms with different types of cancers and found that both polymorphisms are significantly associated with overall cancers [15]. The subgroup analyses indicated 1772 C/T polymorphism in association with decreasing the risk of renal cell carcinoma and the 1790 G/A polymorphism with significantly increased cancer risk in the Asian and Caucasian population [15]. However, a good number of single genetic association (SGA) studies also reported the association of these two polymorphisms with other diseases, including type 2 diabetes (T2D), cardiovascular diseases (CVD), lung disease, autoimmune diseases, inflammatory diseases, preeclampsia, osteoarthritis, lumbar disc degeneration, high altitude polycythemia, age-related macular degeneration and many more [16–54]. The SGA study of Hernández-Molina et al. [18] reported that *HIF1A* 1772 C/T is a significant genetic factor for autoimmune disease, whereas some other studies [25, 31] found its insignificant association. Similarly, some authors showed the significant association of *HIF1A* (1772 C/T and 1790 G/A) with cardiovascular diseases (CVD) [21, 40], though some authors did not find the significant effect in the same question [22, 26]. Again for inflammatory diseases, a significant association was claimed by [20, 38], and an insignificant association by [27, 32, 41]. For Chronic obstructive pulmonary disease (COPD), Yu et al. [17] and Putra et al. [39] claimed the significant and insignificant association with *HIF1A* gene polymorphisms, respectively. Wei et al. [37] showed significant association of 1772 C/T and insignificant association of 1790 G/A polymorphisms of *HIF1A* with COPD. The both SNPs of *HIF1A* gene were significantly associated with preeclampsia [16, 24], but another study found their insignificant association [34]. Likewise, Geza et al. [29] reported the significant association of diabetes (type 1 & 2) with *HIF1A* 1772 C/T polymorphism, and Yamada et al. [35] also suggested that *HIF1A* 1772 C/T is significantly associated with type 2 diabetes (T2D) and *HIF1A* 1790 G/A is not. Another two studies claimed the insignificant association between *HIF1A* gene polymorphisms and type 2 diabetes [45, 50]. Ekberg et al. [51], and Bi et al. [52] both found the significant association of *HIF1A* gene polymorphisms with diabetic complication diseases, but Liu et al. (a) [45] and Pichu et al. (b) [50] found no relation. Also, Lin et al. [33] reported that the *HIF1A* 1790 G/A might be played a protecting role significantly to develop the lumbar disc degeneration (LDD), and *HIF1A* 1772 C/T did not play any role with the severity of LDD. Some authors also checked the association of the *HIF1A* gene with cellulite [28], hemodialysis patients [30], high-altitude polycythemia (HAPC) [36], and age-related macular degeneration (AMD) [23]. They found the significant association of *HIF1A* with cellulite, and insignificant association of *HIF1A* with hemodialysis patients, HAPC and AMD risk.

Thus, we observed that different SGA studies produce inconsistent results about the association of *HIF1A* gene polymorphisms with multiple disease risks beyond cancers. This type of inconsistent results may be produced due to the small sample size and/or heterogeneous population in each of the individual SGA studies. Therefore, a consensus decision about the association of *HIF1A* gene polymorphisms with multiple disease risk is required to make a treatment plan against this genetic effect. To make a consensus decision about the contradictory findings of different studies more accurately, researchers usually consider statistical meta-analysis [15, 55–60]. The meta-analysis makes a decision about the association more accurately compared to SGA studies. Therefore, in this study, we considered statistical meta-analysis to make a consensus decision about the association of *HIF1A* gene (1772 C/T and 1790 G/A) polymorphisms with several disease risks excluding cancers, giving the weight on large sample size and appropriate statistical modeling.

## Material and methods

### Search strategy

PubMed, PubMed Central and Google Scholar were searched to retrieve relevant articles published between 2001 to October 2021 in the English language for this Meta-analysis. For searching the following terminologies were considered: (i) HIF1A, (ii) genetic association, (iii) SNPs, (iv) HIF1A, polymorphisms, (v) rs11549465 or 1772 C/T or P582S, (vi) rs11549467 or 1790 A/G or A588T, (vii) case-control study, (viii) disease, (ix) HIF1A, diseases (x) HIF1A, disorders.

### Eligibility criteria

The title and abstract of the primarily selected relevant studies were independently investigated by two authors. For the final review some important inclusion-exclusion criteria were used to extract data and only included if the studies were (i) designed to examine the association between *HIF1A* gene polymorphisms (C1772T, A1790G) and disease/ disorder risk; (ii) Human case-control studies; (iii) sufficient to provide significant data of genotype frequency.

### Data extraction

For the final review, the following information from each of the included studies was extracted, like; first author, year of publication, country of origin, ethnicity of the study subject, number of cases and control, disease type, allelic and genotypic distribution, and so on according to the PRISMA statement [61]. To confirm the validity of a selected SGA study for inclusion in the meta-analysis, the Hardy-Weinberg equilibrium (HWE) test was performed using the Chi-square statistic. A study was considered suitable for meta-analysis only if Pr{χ^2^_obs_ ≤ χ^2^} ≥ .05 exist (Table 1).

**Table 1:** Characteristic of 38 and 24 studies included in the meta-analysis of *HIF1A* 1772 C/T and 1790 G/A polymorphisms, respectively.

Author	Year	Country	Ethnicity	Diseases	Case/ Control	*P*_HWE_
**rs11549465**						
Harati-Sadegh et al. [16]	2018	Iran	Mixed	Preeclampsia	203/202	0.038
Yu et al. [17]	2017	China	Asian	Chronic obstructive pulmonary disease (COPD)	164/161	0.025
Hernandez-Molina et al. [18]	2017	Mexico	Mixed	Primary Sjogren syndrome	106/135	0.038
Fernandez-Torres et al. [20]	2015	Mexico	Mixed	Osteoarthritis	70/66	0.230
Hlatky et al. [21]	2007	USA	Caucasian	Coronary artery disease (CAD)	909/466	0.157
Duran et al. [22]	2015	Spain	Caucasian	Coronary artery disease (CAD)	518/112	0.994
Okur et al. [23]	2014	Turkey	Caucasian	Age-related macular degeneration (AMD)	87/80	0.779
Andraweera et al. [24]	2014	Sri Lanka	Asian	Preeclampsia	174/168	0.262
Feng et al. [25]	2014	China	Asian	Systemic lupus erythematosus	1495/2294	0.397
Torres et al. [27]	2010	Spain	Caucasian	Gaint cell arteritis	215/470	0.064
Emanuele et al. [28]	2010	Italy	Caucasian	Cellulitis	200/200	0.000
Geza et al. (a) [29]	2009	Hungary	Caucasian	Type 1 diabetes	166/354	0.203
Geza et al. (b) [29]	2009	Hungary	Caucasian	Type 2 diabetes	370/354	0.203
Zheng et al. [30]	2009	Korea	Asian	Hemodialysis	14/360	0.257
Wipff et al. [31]	2009	France	Caucasian	Systemic sclerosis	640/463	0.730
Chachami et al. [32]	2013	Greek	Caucasian	Osteoarthritis	134/63	0.777
Lin et al. [33]	2013	China	Asian	Lumbar disc degeneration (LDD)	274/301	0.193
Nava-Salazar et al. [34]	2011	Mexico	Mixed	Preeclampsia	150/105	0.608
Yamada et al. [35]	2005	Japan	Asian	Type 2 diabetes	440/572	0.084
Chen et al. [36]	2016	China	Asian	High altitude polycythemia (HAPC)	234/250	0.446
Wei et al. [37]	2015	China	Asian	Chronic obstructive pulmonary disease (COPD)	120/112	0.733
de Carvalho Fraga et al. [38]	2013	Brazil	Mixed	Oral lichen planus (OLP)	32/88	0.000
Putra et al. [39]	2013	Japan	Asian	Chronic obstructive pulmonary disease (COPD)	48/110	0.545
Q. Liu et al. [40]	2013	China	Asian	Coronary artery disease (CAD)	356/213	0.862
Zafar et al. [42]	2021	Pakistan	Asian	Metabolic syndrome	200/200	0.031
Sheng et al. [43]	2019	China	Asian	Left ventricular hypertrophy	198/385	0.097
Urganci et al. [44]	2019	Turkey	Asian	Psoriasisÿ	150/150	0.576
Liu et al. (a) [45]	2021	China	Asian	Type 2 diabetes	150/144	0.397
Liu et al. (b) [45]	2021	China	Asian	Diabetic retinopathy	149/144	0.397
Takagi et al. [46]	2020	Japan	Asian	Systemic sclerosis	182/178	0.468
Saravani et al. [47]	2019	Iran	Asian	Multiple sclerosis	150/150	0.014
Qin et al. [48]	2020	China	Asian	Parkinson’s disease	1692/1419	0.483
Tsukatani et al. [49]	2021	Japan	Asian	Pressure injury	130/48	0.883
Pichu et al. (a) [50]	2015	India	Asian	Type 2 diabetes	79/66	0.000
Pichu et al. (b) [50]	2015	India	Asian	Diabetic food ulcer	79/66	0.000
Ekberg et al. [51]	2019	Sweden	Caucasian	Diabetic retinopathy	555/148	0.000
Bi et al. [52]	2015	China	Asian	Diabetic nephropathy	140/104	0.395
Gu et al. [53]	2013	USA	Caucasian	Diabetic nephropathy	571/594	0.159
**rs11549467**						
Harati-Sadegh et al. [16]	2018	Iran	Mixed	Preeclampsia	203/202	0.637
Senhaji et al. [41]	2017	Morocco	Mixed	Inflammatory bowel disease (IBD)	199/308	0.810
Yu et al. [17]	2017	China	Asian	Chronic obstructive pulmonary disease (COPD)	164/161	0.000
Hernandez-Molina et al. [18]	2017	Mexico	Mixed	Primary Sjogren syndrome	108/91	0.958
Fernandez-Torres et al. [20]	2015	Mexico	Mixed	Osteoarthritis	70/66	0.951
Hlatky et al. [21]	2007	USA	Caucasian	Coronary artery disease (CAD)	909/466	0.815
Bahadori et al. [26]	2010	Austria	Caucasian	Peripheral artery disease (PAD)	917/969	0.613
Torres et al. [27]	2010	Spain	Caucasian	Gaint cell arteritis	215/470	0.908
Chachami et al. [32]	2013	Greek	Caucasian	Osteoarthritis	134/63	0.846
Lin et al. [33]	2013	China	Asian	Lumbar disc degeneration (LDD)	274/301	0.062
Nava-Salazar et al. [34]	2011	Mexico	Mixed	Preeclampsia	150/105	0.961
Yamada et al. [35]	2005	Japan	Asian	Type 2 diabetes	440/572	0.364
Chen et al. [36]	2016	China	Asian	High altitude polycythemia (HAPC)	234/250	0.092
Wei et al. [37]	2015	China	Asian	Chronic obstructive pulmonary disease (COPD)	120/112	0.585
Putra et al. [39]	2013	Japan	Asian	Chronic obstructive pulmonary disease (COPD)	48/110	0.655
Q. Liu et al. [40]	2013	China	Asian	Coronary artery disease (CAD)	356/213	0.753
Sheng et al. [43]	2019	China	Asian	Left ventricular hypertrophy	198/385	0.058
Liu et al. (a) [45]	2021	China	Asian	Type 2 diabetes	150/144	0.765
Liu et al. (b) [45]	2021	China	Asian	Diabetic retinopathy	149/144	0.765
Takagi et al. [46]	2020	Japan	Asian	Systemic sclerosis	182/174	0.409
Qin et al. [48]	2020	China	Asian	Parkinson’s disease	1692/1419	0.173
Tsukatani et al. [49]	2021	Japan	Asian	Pressure injury	130/48	0.883
Pichu et al. (a) [54]	2018	India	Asian	Type 2 diabetes	185/145	0.000
Pichu et al. (b) [54]	2018	India	Asian	Diabetic food ulcer	199/145	0.000

*P*_HWE_ *P*-value of the chi-square goodness-of-fit test for Hardy-Weinberg equilibrium in control population; *P*_HWE_ > 0.05 means satisfied HWE, otherwise not

### Quality assessment

Two authors independently checked the assessment of individual study quality by using the Newcastle-Ottawa Scale (NOS) [62]. The total Nine point NOS score was generated through the categories of selection (4 points), comparability (2 points), and exposure (3 points). The NOS score of an individual study is considered poor (0–3), fair (4–6) and excellent (7–9) quality. In our meta-datasets, 38 studies showed excellent quality and 3 were fair quality (S1 Table).

### Statistical meta-analysis

To perform meta-data on SGA studies, we used the following statistical analysis. The HWE test was performed using the Chi-square statistic to confirm the suitability of a selected study for inclusion in the meta-analysis. The consistency of genotypic ratio under the control population was used as the null hypothesis (Ho) for the HWE test. The test statistic of Ho is defined as

$$\chi^{2}=\sum_{i=1}^{3}\frac{{\left(O_{i}-E_{i}\right)}^{2}}{E_{i}}∼\chi_{\left(1\right)}^{2}$$ (1)

which follows chi-square distribution with 1 degree of freedom, where *O*_*i*_ and *E*_*i*_ denote observe and expected frequency of the genotype, respectively. If *p* and *q* denote the probabilities of two alleles (e.g. *C* and *T*), respectively and *O*_*i*_ = *obs(i)* is observed frequency of *i*th genotype among the 3 genotypes *CC*, *CT* and *TT*. Then *p* and *q* are defined as follows

$$p=\frac{2\times obs(CC)+obs(CT)}{2\times (obs(CC)+obs(CT)+obs(TT))};\;and\;q=1-p$$ (2)

Then the expected frequency of *i*th genotype is *E*_*i*_ = *E(i)*, which defined as *E(CC) = p*^*2*^*n*, *E(CT) = 2pqn*, *E(TT) = q*^*2*^*n*, where *n* is total number of observations.

To investigate the association of SNPs with multiple diseases based on pooled odds ratios (ORs), the individual OR of *k*th SGA study was calculated as follows

$${OR}_{k}=\frac{\frac{b_{1k}}{b_{2k}}}{\frac{b_{3k}}{b_{4k}}}=\frac{b_{1k}b_{4k}}{b_{2k}b_{3k}}$$ (3)

where *b*_1*k*_ and *b*_2*k*_ stands for exposures and *b*_3*k*_ and *b*_4*k*_ non-exposures frequencies, in case-control groups of *k*th study, respectively (for example, the genetic model *C* vs. *T*, where *C* is exposer and *T* is non-exposer). Then the pooled ORs under the each of five different genetic models (dominant model [*CC* + *CT* vs. *TT* or *AA* + *AG* vs. *GG*], homozygote model [*CC* vs. *TT* or *AA* vs. *GG*], heterozygote model [*CT* vs. *TT* or *AG* vs. *GG*], recessive model [*CC* vs. *CT* + *TT* or *AA* vs. *AG* + *GG*], and allelic contrast model [*C* vs. *T* or *A* vs. *G*]) was calculated by using the random effects model (REF) for the highly significant heterogeneity (*p-*value < 0.10) among SGA studies, otherwise, fixed effects model (FEM) was used as suggested by other researchers [63, 64]. This heterogeneity was tested using Cochran’s *Q* statistic which will be introduced later. To calculate pooled ORs based on FEM, the Mentel-Haenszel (MH) method was used as follows.

The FEM for *k*th SGA study is defined as

$${\hat{\beta }}_{k}=\beta_{F}+\epsilon_{k},$$ (4)

where,

$${\hat{\beta }}_{F}={\hat{OR}}_{MH}=\frac{\sum_{k=1}^{K}\left(\frac{b_{1k}b_{4k}}{N_{k}}\right)}{\sum_{k=1}^{K}\left(\frac{b_{2k}b_{3k}}{N_{k}}\right)}=\sum_{k=1}^{K}(\frac{\frac{b_{2k}b_{3k}}{N_{k}}}{\sum_{i=1}^{K}\left(\frac{b_{2k}b_{3k}}{N_{k}}\right)})\times {OR}_{k},$$ (5)

$$Var({\hat{\beta }}_{F})=\frac{1}{\sum_{k=1}^{K}\left(\frac{b_{2k}b_{3k}}{N_{k}}\right)},$$

${\hat{\beta }}_{k}=\ln\left({OR}_{k}\right),$ *N*_*k*_ *= b*_1k_*+ b*_2k_ *+ b*_3k_ *+ b*_4k_ and the error term $\epsilon_{k}∼N(0,{\hat{\sigma }}_{k}^{2}).$

Again, to calculate pooled ORs based on REM, the inverse variance method was used as follows.

The REM for *k*th SGA study is defined as

$${\hat{\beta }}_{k}=\beta_{R}+\nu_{k}+\epsilon_{k},$$ (6)

where, $\nu_{k}∼N(0,\tau^{2}),\beta_{R}=\frac{\sum_{k=1}^{K}w_{kR}{\hat{\beta }}_{k}}{\sum_{k=1}^{K}w_{kR}},\;se({\hat{\beta }}_{R})=\sqrt{var({\hat{\beta }}_{R})}=\sqrt{\frac{1}{\sum_{k=1}^{K}w_{kR}}}$, $w_{kR}=\frac{1}{\sigma_{k}^{2}+\tau^{2}},$

$$\tau^{2}=\frac{Q-(K-1)}{\sum w_{k}-(\frac{\sum w_{k}^{2}}{\sum w_{k}})},w_{k}=\frac{1}{{\hat{\sigma }}_{k}^{2}},\;\mathrm{and}$$

$${\hat{\sigma }}_{k}^{2}=var(ln({OR}_{k}))=\frac{1}{b_{1k}}+\frac{1}{b_{2k}}+\frac{1}{b_{3k}}+\frac{1}{b_{4k}}$$ (7)

The 95% confidence interval (CI) for pooled ORs can be obtained based on z-statistic as follows

$$Pro\left\{{\hat{\beta }}_{F}-1.96\sqrt{Var({\hat{\beta }}_{F})}\leq z\leq {\hat{\beta }}_{F}+1.96\sqrt{Var({\hat{\beta }}_{F})}\right\}=0.95,\;\mathrm{for}\;\mathrm{FEM}$$

$$Pro\left\{{\hat{\beta }}_{R}-1.96\sqrt{Var({\hat{\beta }}_{R})}\leq z\leq {\hat{\beta }}_{R}+1.96\sqrt{Var({\hat{\beta }}_{R})}\right\}=0.95,\;\mathrm{for}\;\mathrm{REM}$$

where

$$z=\left\{ \begin{array}{c} \frac{\sum_{k}w_{k}{\hat{\beta }}_{k}}{\sqrt{\sum_{k}w_{k}}},\;\mathrm{for}\;\mathrm{FEM}\\ \frac{\sum_{k}w_{kR}{\hat{\beta }}_{k}}{\sqrt{\sum_{k}w_{kR}}},\;\mathrm{for}\;\mathrm{REM} \end{array}\right.$$ (8)

Then the Cochran’s *Q* statistic [65] is defined as

$$Q=\sum_{k=1}^{K}w_{k}{\left({\hat{\beta }}_{k}-\frac{\sum_{k=1}^{K}w_{k}{\hat{\beta }}_{k}}{\sum_{k=1}^{K}w_{k}}\right)}^{2}∼\chi_{\left(K-1\right)}^{2}$$ (9)

and its extended Higgin’s and Thompson *I*^*2*^—statistic [66] was also used to check the heterogeneity of SGA studies. The *I*^*2*^-statistic is defined as

$$I^{2}=\max\left\{0,\;\frac{Q-(K-1)}{Q}\times 100\%\right\}$$ (10)

The *I*^*2*^ values >25%, >50% and >75% defined as low, moderate, and high heterogeneity, respectively.

Subgroup analyses were performed based on ethnicity and disease types. Sensitivity analysis was carried out using both the full data and reduced data, where the reduced dataset did not included the SGA studies that were rejected by the HWE validation test.

To investigate the publication bias on the included SGA studies in the meta-analysis, we constructed the funnel plot, where the standard error (se) of the estimated effect was plotted against the ORs [63, 64, 67]. Also, Egger’s regression test and Begg’s test [68, 69] was performed for quantitative evaluation (*p-*value < 0.05 indicates the existence of publication bias). The Egger regression test was performed under H_0_: *α* = 0 (absence of publication bias) and the test statistic follows as

$$T=\frac{\hat{ϴ}}{se(\hat{ϴ})}∼t_{\left(K-2\right)}$$ (11)

where $\hat{ϴ}$ is estimated by the least square estimation with the respective following models

$${\hat{\beta }}_{k}\sqrt{w_{k}}=ϴ+\mu \sqrt{w_{k}}+\varepsilon_{k},\;\mathrm{for}\;\mathrm{FEM},\;\mathrm{and}$$ (12)

$${\hat{\beta }}_{k}\sqrt{w_{kR}}=ϴ+\mu \sqrt{w_{kR}}+\varepsilon_{k},\;\mathrm{for}\;\mathrm{REM},$$ (13)

with *ε*_*k*_~*iid N*(0, *σ*^2^). The Begg’s test was performed under H_0_: *α* = 0 (absence of publication bias) and the test statistic follows

$$Z=\frac{C-D}{\sqrt{K(K-1)(2K+5)/18}}∼N(0,1)$$ (14)

where *C* and *D* represents concordant and discordant number, respectively, and obtained by using the Kendall’s ranking of $t_{k}^{*}$ and ${\hat{\sigma }}_{k}^{2}$ or ${\hat{\sigma }}_{kR}^{2}$. Here

$$t_{k}^{*}=\frac{t_{k}-\bar{t}}{\sqrt{\vartheta_{k}^{*}}}$$ (15)

where, *t*_*k*_ = OR_*k*_ is denoted the OR of *k*th study, and

$$\bar{t}=\left\{ \begin{array}{c} \frac{\sum_{k}w_{k}t_{k}}{\sqrt{\sum_{k}w_{k}}},\;\mathrm{for}\;\mathrm{FEM}\\ \frac{\sum_{k}w_{kR}t_{k}}{\sqrt{\sum_{k}w_{kR}}},\;\mathrm{for}\;\mathrm{REM} \end{array}\right.$$ (16)

ϑk*={σ^k2−1∑wk,forFEMσ^kR2−1∑wkR,forREM (17)

Also, we studied a false positive report probability (FPRP) to verify whether the findings could be regarded as false positives or not [70]. We computed the statistical power and FPRP based on our significant ORs using the following mechanism,

FPRP=α(1–π)/[α(1–π)+(1−β)π] (18)

where, α is the level of significance, π is the prior probability and (1- β) is statistical power.

To implement all the statistical analysis, we used ‘meta’ package in R program (http://meta-analysis-with-r.org/).

## Results

### Study characteristics

**Fig 1:**
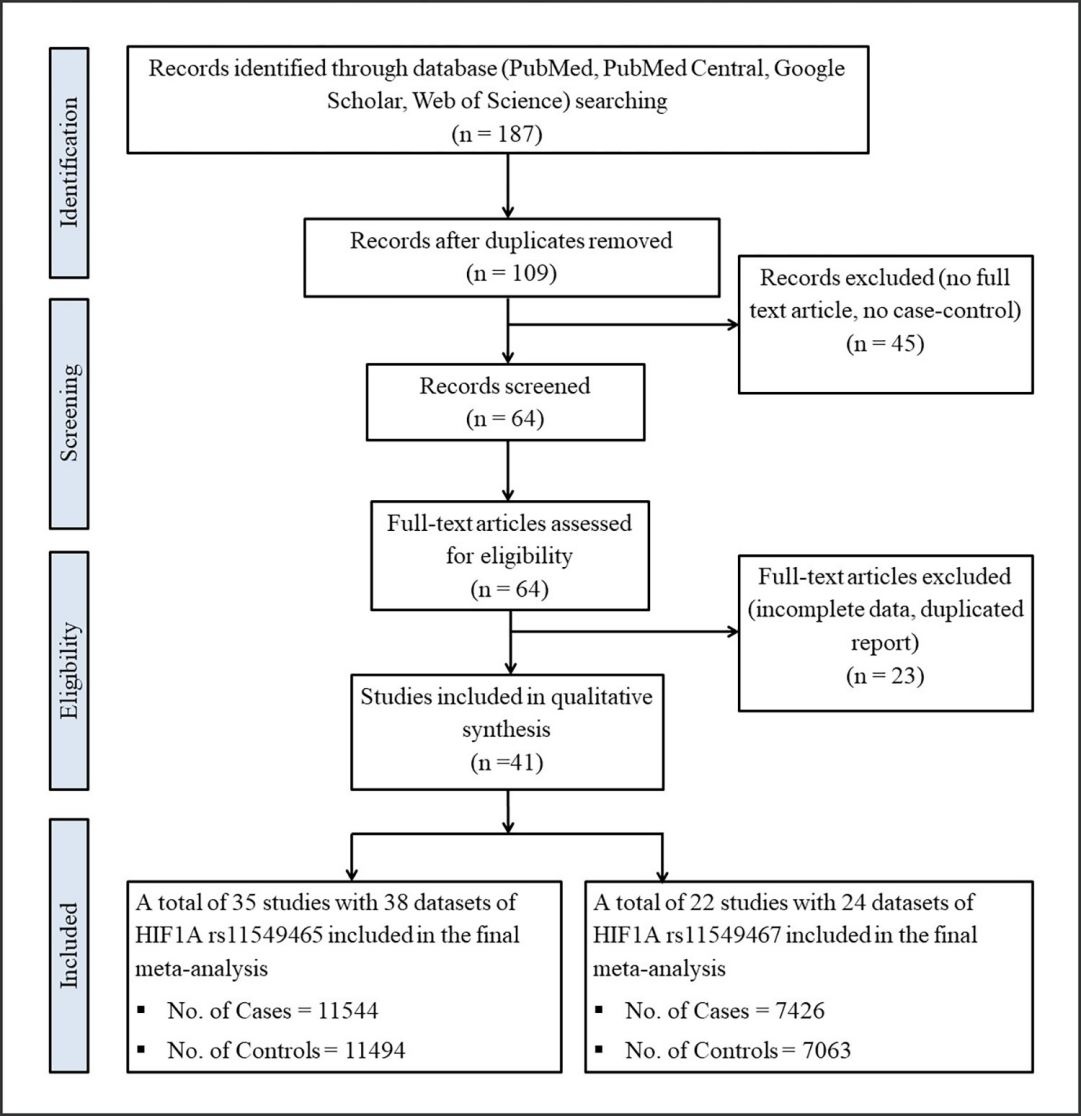
Flow diagram for study selection with *HIF1A* gene polymorphisms rs11549465 and rs11549467.

Initially, 187 studies were selected through text mining that included the *HIF1A* gene and polymorphisms in their title or abstracts. After screening of the duplications, excluding the studies that did not match with the eligibility criteria or had incomplete information, a total of 41 studies were selected based on the PRISMA statement for the final review (Fig 1). In this study, 35 studies comprised 38 datasets of the *HIF1A* 1772 C/T polymorphism with a sample size of 23038 (comprising 11544 cases and 11494 controls), and 22 studies comprised 24 datasets for the *HIF1A* 1790 G/A polymorphism with a sample size of 14489 (comprising 7426 cases and 7063 controls) were incorporated. For Meta-analysis of *HIF1A* 1772 C/T and 1790 G/A polymorphisms, the types of diseases included (after excluding all types of cancer) were grouped as cardiovascular diseases (CVDs), type 2 diabetes (T2D), autoimmune diseases, inflammatory diseases, chronic obstructive pulmonary disease (COPD), preeclampsia, skin disease, diabetic complications, and other (age-related macular degeneration (AMD), Hemodialysis, lumbar disc degeneration (LDD), high altitude polycythemia (HAPC), metabolic syndrome, pressure injury). The ‘other’ disease group was made in case of a single study of each disease to perform this meta-analysis. The subgroup of respective diseases was shown in S4 Table.

#### Quantitative synthesis of *HIF1A* 1772 C/T polymorphism

**Fig 2:**
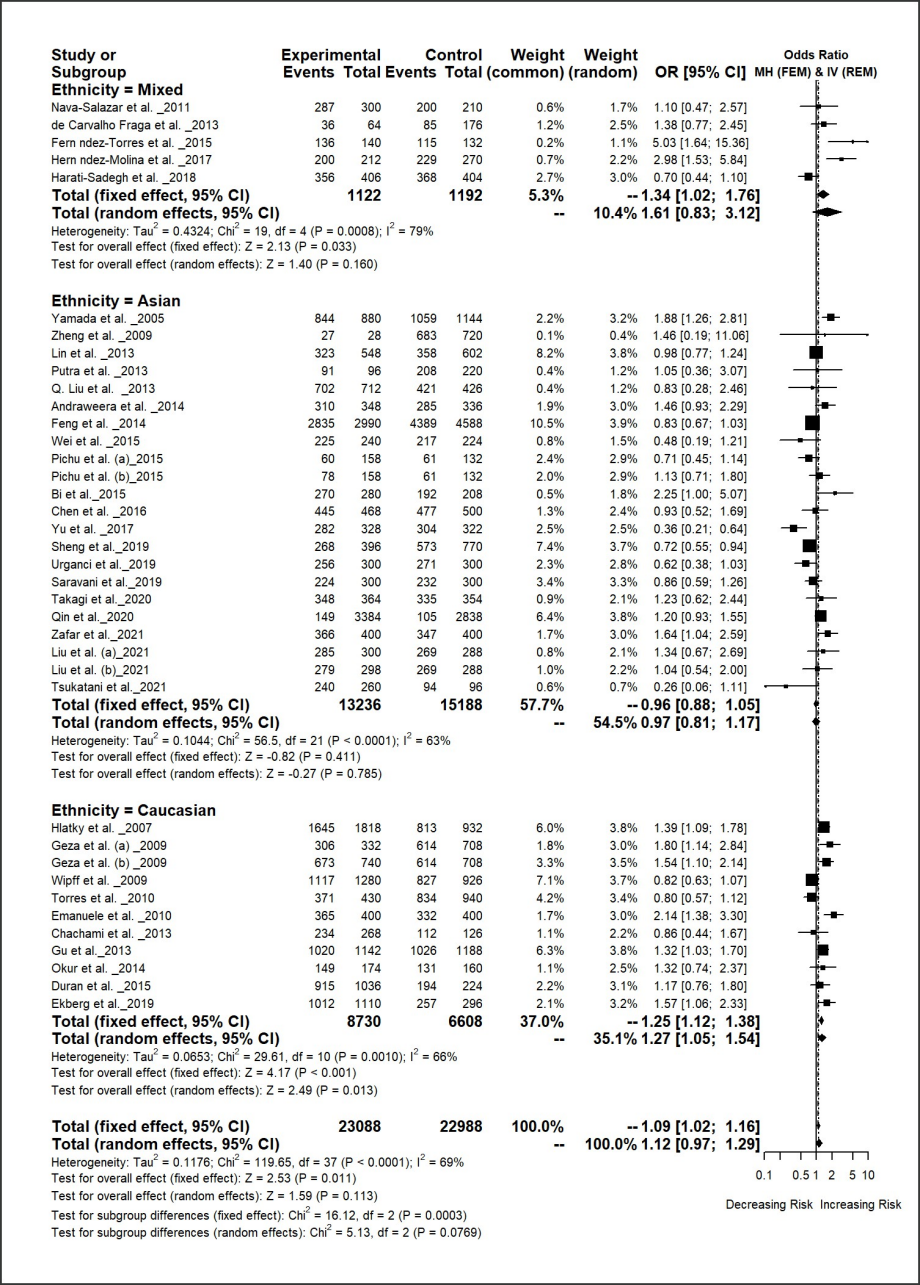
Forest plot of *HIF1A* 1772 C/T polymorphism and overall disease risk for different ethnic populations under allelic model [*C* vs. *T*]. In the forest plot, the square of the horizontal line represents the individual study-specific odds ratios (ORs) with 95% confidence intervals (CIs) and the black area of the squares represents the corresponding study weight. The black diamond reflects the pooled OR and the lateral points of the diamond represent the CI of the overall analyses. The solid vertical lines are the OR of 1 indicates no effect. The dashed vertical line shows the corresponding pooled OR of the analyses.

Results generated through this meta-analysis indicated that the *HIF1A* 17772 C/T polymorphism was insignificantly associated with the overall disease risk under all genetic models [*C* vs. *T*: OR = 1.12, 95% CI = 0.97–1.29, *p-*value = 0.113]; [*CC* vs. *TT*: OR = 1.16, 95% CI = 0.94–1.44, *p-*value = 0.154]; [*CT* vs. *TT*: OR = 1.15, 95% CI = 0.83–1.59, *p-*value = 0.395]; [*CC* + *CT* vs. *TT*: OR = 1.14, 95% CI = 0.86–1.51, *p-*value = 0.375]; and [*CC* vs. *CT* + *TT*: OR = 1.10, 95% CI = 0.93–1.31, *p-*value = 0.257;] (Table 2; Fig 2; S1-S5 Figs in S1 File).

**Table 2:** Meta-analysis of the *HIF1A* rs11549465 C/T and T/C polymorphisms in association with different diseases.

Subgroup	Study number	C vs. T		CC vs. TT		CT vs. TT		CC + CT vs. TT		CC vs. CT + TT	
		OR (95% CI)	*p*-val	OR (95% CI)	*p*-val	OR (95% CI)	*p*-val	OR (95% CI)	*p*-val	OR (95% CI)	*p*-val
**Overall**	38	1.12 [0.97; 1.29]	0.113	1.16 [0.94; 1.44]	0.154	1.15 [0.83; 1.59]	0.395	1.14 [0.86; 1.51]	0.375	1.10 [0.93; 1.31]	0.257
**Preeclampsia**	3	1.03 [0.62; 1.70]	0.911	0.70 [0.26; 1.91]	0.483	0.78 [0.27; 2.29]	0.649	0.70 [0.26; 1.92]	0.491	1.07 [0.77; 1.48]	0.694
**Chronic obstructive pulmonary disease (COPD)**	3	**0.46 [0.30; 0.71]**	**0.000**	0.59 [0.13; 2.67]	0.492	1.52 [0.31; 7.43]	0.603	0.68 [0.15; 3.09]	0.620	**0.43 [0.27; 0.67]**	**0.000**
**Autoimmune disease**	7	1.08 [0.75; 1.56]	0.672	1.02 [0.62; 1.67]	0.938	1.15 [0.68; 1.95]	0.597	1.05 [0.64; 1.72]	0.842	1.08 [0.72; 1.64]	0.703
**Inflammatory disease**	5	1.19 [0.80; 1.77]	0.386	1.40 [0.60; 3.28]	0.437	0.41 [0.05; 3.06]	0.383	0.60 [0.15; 2.34]	0.458	2.95 [0.56; 15.54]	0.201
**Cardiovascular disease (CVD)**	4	1.03 [0.71; 1.48]	0.887	1.18 [0.46; 3.00]	0.733	1.09 [0.64; 1.85]	0.749	1.07 [0.64; 1.79]	0.799	0.99 [0.66; 1.50]	0.978
**Skin disease**	2	0.83 [0.11; 6.59]	0.863	**3.01 [1.09; 8.32]**	**0.034**	1.68 [0.56; 5.11]	0.357	**2.71 [0.98; 7.49]**	**0.055**	0.81 [0.09; 6.85]	0.844
**Type 2 diabetes**	4	1.31 [0.85; 2.00]	0.218	2.06 [0.47; 9.08]	0.340	1.65 [0.84; 3.26]	0.147	2.05 [0.53; 7.96]	0.301	1.33 [0.90; 1.95]	0.148
**Diabetic complications**	5	**1.34 [1.12; 1.61]**	**0.001**	1.59 [0.94; 2.69]	0.085	**2.43 [1.41; 4.18]**	**0.001**	**2.11 [1.29; 3.43]**	**0.003**	1.24 [0.88; 1.75]	0.216
**Others**	5	1.10 [0.91; 1.32]	0.309	0.95 [0.59; 1.52]	0.822	0.95 [0.61; 1.50]	0.841	0.96 [0.62; 1.48]	0.852	1.18 [0.93; 1.49]	0.179
**Ethnicity**											
**Asian**	22	0.97 [0.81; 1.17]	0.785	0.87 [0.66; 1.14]	0.305	1.19 [0.99; 1.45]	0.067	1.10 [0.92; 1.32]	0.300	0.92 [0.73; 1.15]	0.455
**Caucasian**	11	**1.27 [1.05; 1.54]**	**0.013**	**2.00 [1.40; 2.87]**	**0.000**	**1.64 [1.12; 2.40]**	**0.011**	**1.93 [1.35; 2.77]**	**0.000**	**1.24 [1.02; 1.52]**	**0.032**
**Mixed**	5	1.61 [0.83; 3.12]	0.160	0.92 [0.37; 2.30]	0.866	0.18 [0.03; 1.31]	0.091	**0.24 [0.11; 0.54]**	**0.001**	3.38 [0.79; 14.41]	0.100
**HWE tested data**											
**Overall**	28	1.11 [0.97; 1.27]	0.144	1.19 [0.99; 1.55]	0.201	1.19 [0.98; 1.43]	0.072	1.20 [0.99; 1.44]	0.058	1.10 [0.94; 1.28]	0.255

OR (95% CI) is Odds Ratio (95% Confidence Interval); The bold results indicates the statistical significance.

The subgroup analyses results based on disease type showed that the *HIF1A* 1772 C/T polymorphism is significantly associated with increasing the risk of diabetic complications under three genetic models: [*C* vs. *T*: OR = 1.34, 95% CI = 1.12–1.61, *p-*value = 0.001]; [*CT* vs. *TT*: OR = 2.43, 95% CI = 1.41–4.18, *p-*value = 0.001]; [*CC* + *CT* vs. *TT*: OR = 2.11, 95% CI = 1.29–3.43, *p-*value = 0.003]. For skin diseases group, this polymorphism was also significantly increasing the risk of disease under two genotypic models [*CC* vs. *TT*: OR = 3.01, 95% CI = 1.09–8.32, *p-*value = 0.034] and [*CC* + *CT* vs. *TT*: OR = 2.71, 95% CI = 0.99–7.49, *p-*value = 0.055]. Interestingly, the polymorphism significantly decreasing the risk of chronic obstructive pulmonary disease (COPD) under two genetic models [*C* vs. *T*: OR = 0.46, 95% CI = 0.30–0.71, *p-*value = 0.000] and [*CC* vs. *CT* + *TT*: OR = 0.43, 95% CI = 0.27–0.67, *p-*value = 0.000]. However, the subgroup analyses of autoimmune diseases, inflammation, preeclampsia, CVD, T2D and other showed insignificant association with the *HIF1A* 1772 C/T polymorphism.

The subgroup analyses by ethnicity for the *HIF1A* 1772 C/T polymorphism exhibited that this polymorphism was strongly associated with overall disease risk in Caucasian populations under all genetic models [*C* vs. *T*: OR = 1.27, 95% CI = 1.05–1.54, *p-*value = 0.013]; [*CC* vs. *TT*: OR = 2.00, 95% CI = 1.40–2.87, *p-*value = 0.000]; [*CT* vs. *TT*: OR = 1.64, 95% CI = 1.12–2.40, *p-*value = 0.011]; [*CC* + *CT* vs. *TT*: OR = 1.93, 95% CI = 1.35–2.77, *p-*value = 0.000]; and [*CC* vs. *CT* + *TT*: OR = 1.24, 95% CI = 1.02–1.52, *p-*value = 0.032]. This polymorphism showed a low significant association with overall disease risk in mixed population under dominant model [*CC* + *CT* vs. *TT*: OR = 0.24, 95% CI = 0.11–0.54, *p-*value = 0.001] and insignificant association for Asian patients.

#### Sources of heterogeneity

According to the results of heterogeneity analysis, we found the significant heterogeneity of *HIF1A* 1772 C/T polymorphism with overall disease risk under the four genetic models: [*C* vs. *T*: Q = 119.65, df = 37, *p-*value = 0.0001, I^2^ = 69.1%]; [*CT* vs. *TT*: Q = 55.55, df = 37, *p*-value = 0.026, I^2^ = 33.4%]; [*CC* + *CT* vs. *TT*: Q = 48.97, df = 37, *p-*value = 0.090, I^2^ = 24.4%]; and [*CC* vs. *CT* + *TT*: Q = 128.09, df = 37, *p-*value = 0.0001, I^2^ = 71.1%]. Also in subgroup analysis, some genetic model showed the significant heterogeneity in cases of autoimmune disease, inflammatory disease, CVD, skin disease, T2D disease group and Asian, Caucasian and mixed ethnic populations (S2 Table). That subgroup may be the main sources of heterogeneity for conducting the meta-analysis of *HIF1A* 1772 C/T polymorphism.

### Quantitative synthesis of *HIF1A* 1790 G/A polymorphism

**Fig 3:**
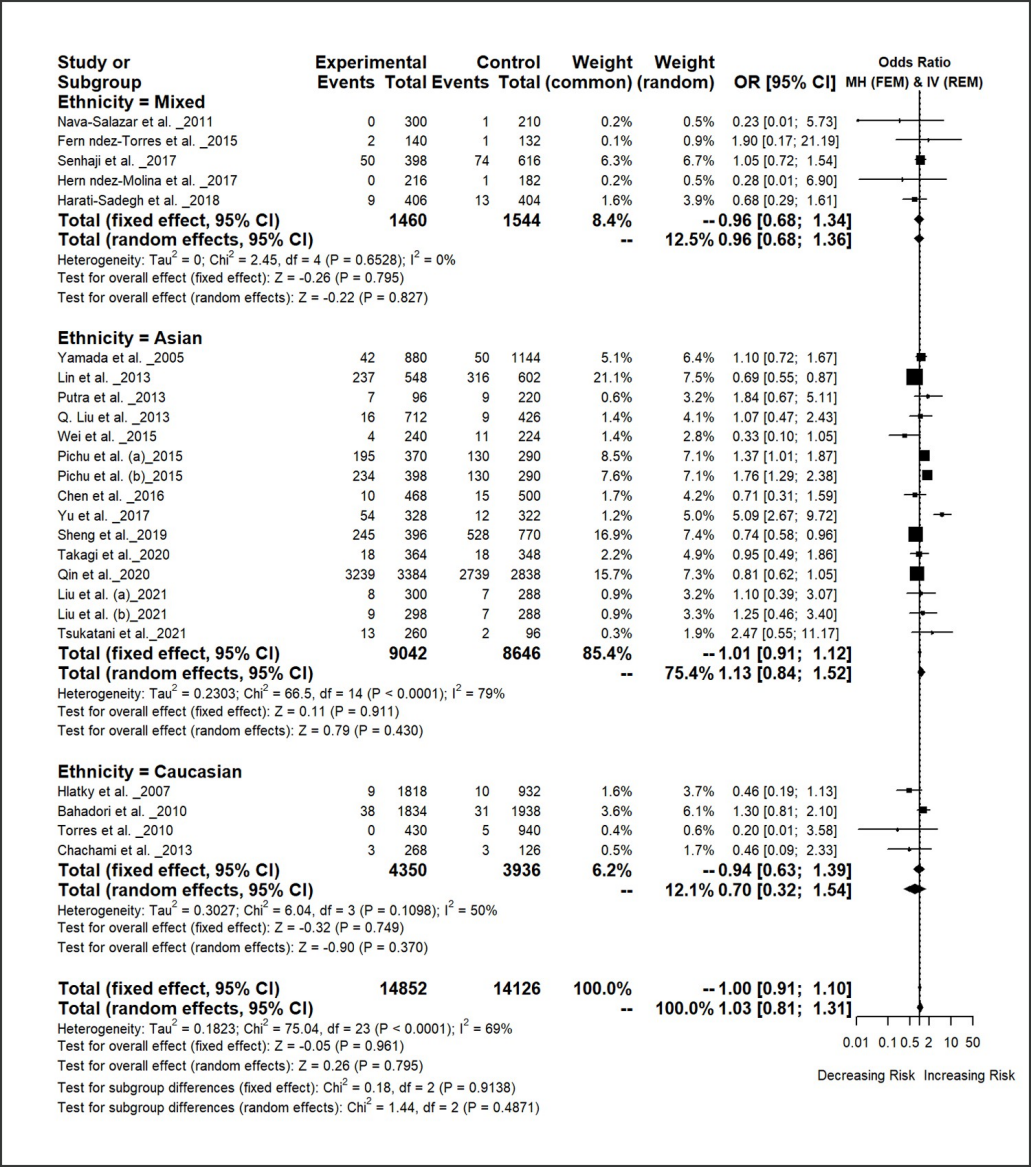
Forest plot of *HIF1A* 1790 G/A polymorphism and overall disease risk for the different ethnic populations under allelic model [*A* vs. *G*].

The pooled estimate of *HIF1A* 1790 G/A polymorphism showed a significant association with decrease the risk of overall disease under recessive model [*AA* vs. *GA* + *GG*: OR = 0.78, 95% CI = 0.67–0.91, *p-*value = 0.002]. (Table 3; Fig 3; S6-S10 Figs in S1 File).

**Table 3:** Summary results of ORs and 95% CI of *HIF1A* rs11549467 G/A polymorphism association with diseases.

Subgroup	Study number	A vs. G		AA vs. GG		AG vs. GG		AA + AG vs. GG		AA vs. AG + GG	
		OR (95% CI)	*p*-val	OR (95% CI)	*p*-val	OR (95% CI)	*p*-val	OR (95% CI)	*p*-val	OR (95% CI)	*p*-val
**Overall**	24	1.03 [0.81; 1.31]	0.795	0.96 [0.75; 1.23]	0.753	1.18 [0.8; 1.72]	0.402	1.10 [0.79; 1.53]	0.572	**0.78 [0.67; 0.91]**	**0.002**
**Preeclampsia**	2	0.63 [0.27; 1.43]	0.269	NA [NA; NA]	NA	0.62 [0.27; 1.43]	0.263	0.62 [0.27; 1.43]	0.263	0.84 [0.05; 13.43]	0.900
**Inflammatory disease**	5	0.86 [0.70; 1.06]	0.166	0.99 [0.21; 4.63]	0.992	0.98 [0.65; 1.46]	0.904	0.98 [0.66; 1.44]	0.904	0.81 [0.62; 1.05]	0.106
**Chronic obstructive pulmonary disease (COPD)**	3	1.54 [0.32; 7.34]	0.588	2.61 [0.51; 13.29]	0.249	1.73 [0.29; 10.55]	0.550	1.67 [0.30; 9.41]	0.562	1.82 [0.43; 7.79]	0.417
**Autoimmune disease**	2	0.90 [0.47; 1.72]	0.742	0.13 [0.00; 6.57]	0.308	1.00 [0.50; 1.98]	0.996	0.94 [0.48; 1.86]	0.869	0.45 [0.04; 5.09]	0.522
**Cardiovascular disease (CVD)**	4	0.83 [0.67; 1.02]	0.080	**0.46 [0.25; 0.84]**	**0.012**	0.82 [0.50; 1.35]	0.441	0.79 [0.47; 1.34]	0.385	0.73 [0.52; 1.03]	0.076
**Type 2 diabetes**	3	1.26 [0.99; 1.60]	0.062	1.48 [0.89; 2.46]	0.128	1.86 [0.71; 4.85]	0.207	1.54 [0.85; 2.79]	0.155	0.79 [0.50; 1.23]	0.299
**Diabetic complications**	2	**1.71 [1.27; 2.28]**	**0.000**	**2.34 [1.40; 3.89]**	**0.001**	2.83 [0.65; 12.31]	0.166	2.30 [0.89; 5.96]	0.087	1.07 [0.69; 1.66]	0.759
**Others**	3	**0.72 [0.58; 0.89]**	**0.003**	**0.50 [0.32; 0.78]**	**0.002**	0.82 [0.58; 1.15]	0.246	**0.72 [0.52; 0.99]**	**0.042**	**0.60 [0.41; 0.87]**	**0.008**
**Ethnicity**											
**Asian**	15	1.13 [0.84; 1.52]	0.430	0.94 [0.49; 1.80]	0.850	1.47 [0.92; 2.37]	0.111	1.30 [0.86; 1.99]	0.217	**0.78 [0.67; 0.91]**	**0.002**
**Caucasian**	4	0.94 [0.63; 1.39]	0.749	4.27 [0.06; 294.6]	0.502	0.57 [0.21; 1.54]	0.268	0.63 [0.26; 1.52]	0.308	1.17 [0.19; 7.34]	0.867
**Mixed**	5	0.96 [0.68; 1.34]	0.795	0.79 [0.15; 4.15]	0.785	0.97 [0.67; 1.41]	0.869	0.96 [0.67; 1.39]	0.831	0.81 [0.22; 2.93]	0.747
**HWE tested data**											
**Overall**	21	**0.83 [0.74; 0.93]**	**0.001**	0.96 [0.75; 1.23]	0.753	0.92 [0.78; 1.08]	0.295	0.87 [0.74; 1.02]	0.091	**0.73 [0.61; 0.87]**	**0.001**

OR (95% CI) is Odds Ratio (95% Confidence Interval); The bold results indicate the statistical significance.

The subgroup analyses based on disease type showed that the *HIF1A* 1790 G/A polymorphism is significantly associated with increasing the risk of diabetic complications under the allelic contrast model [*A* vs. *G*: OR = 1.71, 95% CI = 1.27–2.28, *p-*value = 0.000] and homozygote model [*AA* vs. *GG*: OR = 2.34, 95% CI = 1.40–3.89, *p-*value = 0.001]. This polymorphism also significantly associated with decreasing the risk of CVD under homozygote model [*AA* vs. *GG*: OR = 0.46, 95% CI = 0.25–0.84, *p-*value = 0.012] and other’ disease group under four genetic models [*A* vs. *G*: OR = 0.72, 95% CI = 0.58–0.89, *p-*value = 0.003]; [*AA* vs. *GG*: OR = 0.50, 95% CI = 0.32–0.78, *p-*value = 0.002]; [*AA* + *AG* vs. *GG*: OR = 0.72, 95% CI = 0.52–0.99, *p-*value = 0.042]; [*AA* vs. *AG* + *GG*: OR = 0.60, 95% CI = 0.41–0.87, *p-*value = 0.008] (Table 3).

The subgroup analyses by ethnicity of the *HIF1A* 1790 G/A polymorphism indicated that in the Asian population this polymorphism was significantly associated with decreasing overall disease risk under the recessive model [*AA* vs. *GA* + *GG*: OR = 0.78, 95% CI = 0.67–0.91, *p-*value = 0. 002]. However, this polymorphism revealed an insignificant association with overall disease risk for the Caucasian and mixed populations (Table 3).

### Sources of heterogeneity

In this Meta-analysis, significant heterogeneity was observed in different studies of *HIF1A* 1790 G/A polymorphism for overall analysis under three genetic models [*A* vs. *G*: Q = 75.04, df = 23, *p-*value = 0.0001, I^2^ = 69.4%]; [*GA* vs. *GG*: Q = 99.55, df = 23, *p-*value = 0.0001, I^2^ = 76.9%]; [*AA* + *GA* vs. *GG*: Q = 89.84, df = 23, *p-*value = 0.0001, I^2^ = 74.4%]. The subgroup analysis suggested that some genetic model showed significant heterogeneity in the cases of COPD, CVD, T2D, Diabetic complications, Asian, and Caucasian populations. (S2 Table).

### Publication bias checking

**Fig 4:**
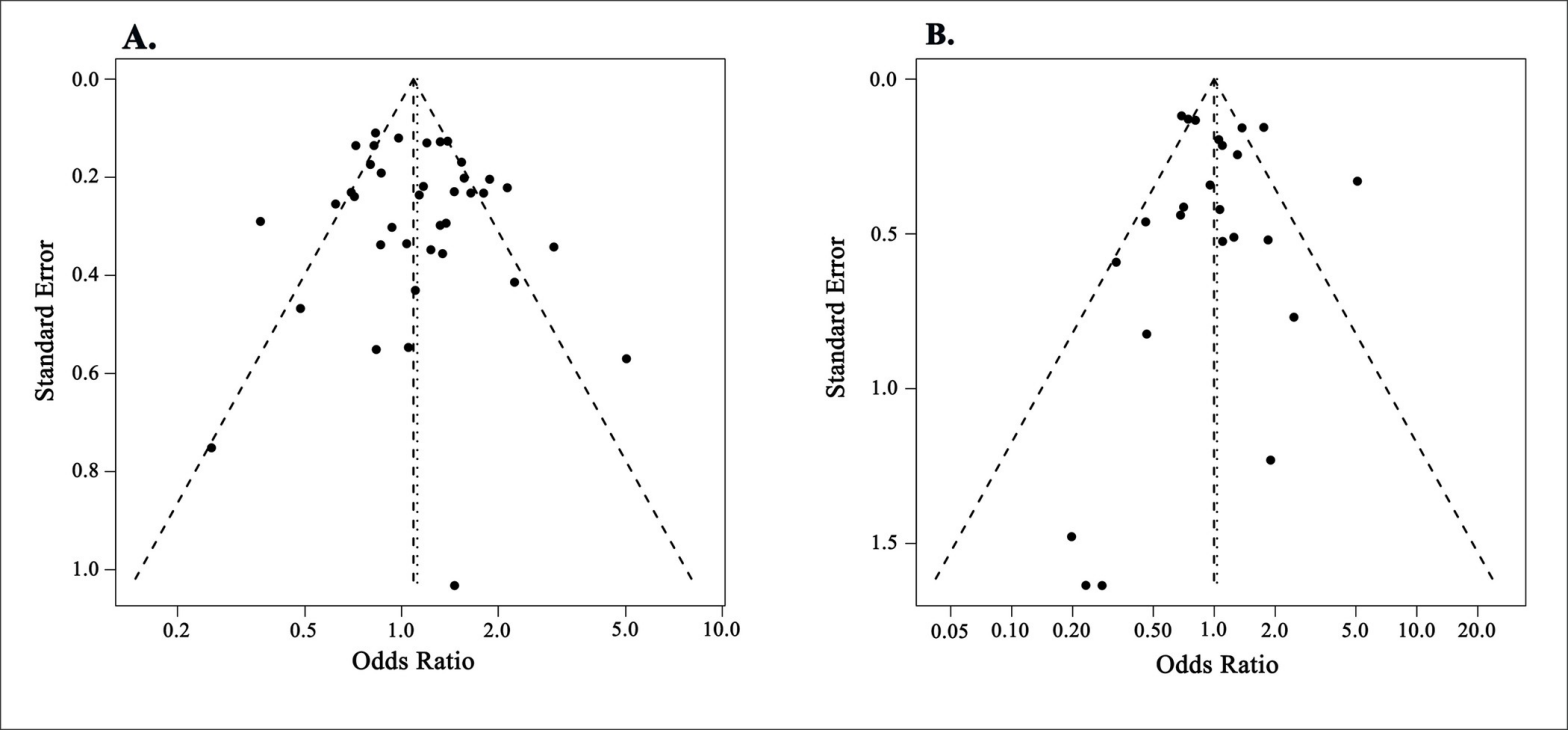
Funnel plot for publication bias checking of *HIF1A* variants (a) 1772 C/T for *C* allele vs. *T* allele (b) 1790 G/A for *A* allele vs. *G* allele.

The funnel plot was used to check publication bias of *HIF1A* gene 1772 C/T, and 1790 G/A polymorphisms with allelic model *C* vs. *T*, and *A* vs. *G*, respectively. The conventionally constructed plots confirmed the symmetric distribution of ORs based on standard error and suggested no evidence of publication bias (Fig 4). Also, the Begg’s test and Egger’s linear regression test analysis data confirmed no significant publication bias under the allelic model of *HIF1A* 1772 C/T polymorphism [*C* vs. *T* allele; *p-*value = 0.9900 and 0.5052 respectively], and for the 1790 G/A [*A* vs. *G* allele; *p-*value = 0.7284 and 0.8537 respectively] polymorphisms (S3 Table).

### Sensitivity analysis

In this study, sensitivity analysis was performed to increase the reliability of meta-analysis results. Studies that do not qualify HWE were excluded to investigate the existence of the attained results. The statistical associations of the results were not altered after excluding the respective studies, which confirmed the reliability of this meta-analysis (Tables 2 and 3).

### False positive report probability (FPRP) and power analyses

We performed false-positive report probability (FPRP) to assess whether associations reported previously were false positives. We preset FPRP at 0.2 as the threshold for biological importance and a prior probability ‘π’ at 0.01 to detect the significant OR [71]. We computed the statistical power and FPRP by fixing the odds ratio at 1.5 (or, 1/1.5 for protective effect) for identifying important biologic effects [70]. It should be mentioned here that an OR value at 1.5 is considered as a plausible value for a significant biologic effects [72, 73]. The association was considered significant, when the FPRP value was less than 0.2 [74]. Based on the above discussion, the rs11549465 SNP significantly increased the overall disease risk in Caucasian patients. Also, the rs11549465 SNP significantly increased the risk of diabetic complications and decreasing the risk of COPD (Table 4). The rs11549467 SNP significantly decreased the overall disease risk for Asian patients and subgroup of CVD risk (Table 4).

**Table 4:** Results of false positive report probability analysis for significant findings.

Genotype and Variables	OR (95% CI)	Statistical Power^a^	FPRP values for prior probabilities at					
			0.25	0.1	0.01	0.001	0.0001	0.00001
rs11549465 and Caucasian								
C vs. T	1.27 [1.05; 1.54]	0.955	0.045^b^	0.125^b^	0.610	0.940	0.994	0.999
CC vs. TT	2.00 [1.40; 2.87]	0.986	0.001^b^	0.002^b^	0.017^b^	0.146^b^	0.631	0.945
CT vs. TT	1.64 [1.12; 2.40]	0.323	0.092^b^	0.233	0.769	0.971	0.997	1.000
CC + CT vs. TT	1.93 [1.35; 2.77]	0.992	0.001^b^	0.003^b^	0.035^b^	0.267	0.785	0.973
CC vs. CT + TT	1.24 [1.02; 1.52]	0.967	0.106^b^	0.263	0.797	0.975	0.997	1.000
rs11549465 and Chronic obstructive pulmonary disease (COPD)								
C vs. T	0.46 [0.30; 0.71]	0.933	0.001^b^	0.004^b^	0.046^b^	0.327	0.829	0.980
CC vs. CT + TT	0.43 [0.27; 0.67]	0.879	0.001^b^	0.002^b^	0.021^b^	0.179^b^	0.685	0.956
rs11549465 and Skin disease								
CC vs. TT	3.01 [1.09; 8.32]	0.497	0.163^b^	0.378	0.870	0.985	0.999	1.000
CC + CT vs. TT	2.71 [0.98; 7.49]	0.578	0.221	0.460	0.903	0.990	0.999	1.000
rs11549465 and Diabetic complications								
C vs. T	1.34 [1.12; 1.61]	0.886	0.006^b^	0.018^b^	0.166^b^	0.667	0.953	0.995
CT vs. TT	2.43 [1.41; 4.18]	0.777	0.005^b^	0.015^b^	0.145^b^	0.632	0.945	0.994
CC + CT vs. TT	2.11 [1.29; 3.43]	0.922	0.008^b^	0.025^b^	0.218	0.738	0.966	0.996
rs11549467 and Overall								
AA vs. AG + GG	0.78 [0.67; 0.91]	0.973	0.005^b^	0.014^b^	0.139^b^	0.619	0.942	0.994
rs11549467 and Asian								
AA vs. AG + GG	0.78 [0.67; 0.91]	0.973	0.005^b^	0.014^b^	0.139^b^	0.619	0.942	0.994
rs11549467 and Cardiovascular disease (CVD)								
AA vs. GG	0.46 [0.25; 0.84]	0.860	0.039^b^	0.107^b^	0.569	0.930	0.993	0.999
rs11549467 and Diabetic complications								
A vs. G	1.71 [1.27; 2.28]	0.857	0.001^b^	0.003^b^	0.029^b^	0.231	0.750	0.968
AA vs. GG	2.34 [1.40; 3.89]	0.831	0.004^b^	0.011^b^	0.111^b^	0.557	0.926	0.992
rs11549467 and Others								
A vs. G	0.72 [0.58; 0.89]	0.747	0.009^b^	0.028^b^	0.240	0.761	0.970	0.997
AA vs. GG	0.50 [0.32; 0.78]							
AA + AG vs. GG	0.72 [0.52; 0.99]	0.671	0.162^b^	0.367	0.864	0.985	0.998	1.000
AA vs. AG + GG	0.60 [0.41; 0.87]	0.280	0.070^b^	0.185^b^	0.713	0.962	0.996	1.000

^a^Statistical power was calculated using the number of observations in each subgroup and the corresponding ORs nad *P* values in this table.

^b^The level of false-positive report probability threshold was set at 0.2 and noteworthy findings are presented

## Discussion and conclusion

We performed a statistical meta-analysis to investigate the association of *HIF1A* gene polymorphisms with multiple diseases risks more accurately compare to SGA studies. This analysis was performed based on 41 SGA study’s findings, where the polymorphisms rs11549465 (1772 C/T) and rs11549467 (1790 G/A) of *HIF1A* gene were analyzed based on 11544 and 7426 cases and 11494 and 7063 control samples, respectively. This study included different types of diseases (i.e. CVD, T2D, autoimmune diseases, inflammatory diseases, COPD, preeclampsia, parkinson disease, diabetic complications, AMD, Hemodialysis, LDD, HAPC, metabolic syndrome, and pressure injury) and ethnic groups (i.e. Asian, Caucasian and mixed) were considered in this meta-analysis. The allelic alterations in different ethnic population and their association with diseases were carefully evaluated using five different genetic models (i) dominant models: *CC* + *CT* vs. *TT* or *AA* + *AG* vs. *GG*, (ii) homozygote models: *CC* vs. *TT* or *AA* vs. *GG*, (iii) heterozygote models: *CT* vs. *TT* or *AG* vs. *GG*, (iv) recessive models: *CC* vs. *CT* + *TT* or *AA* vs. *AG* + *GG*, and (v) allelic contrast models: *C* vs. *T* or *A* vs. *G*, for each of 1772 C/T and 1790 G/A polymorphisms. The results of this study suggested that the *HIF1A* 1772 C/T polymorphism is insignificantly associated with overall disease risks under all the genetic models, which indicates that the *C* allele is not associated with overall diseases. The *HIF1A* 1790 G/A polymorphism showed a significant association with overall disease under the recessive model (*AA* vs. *AG* + *GG*), which indicates that the *A* allele is associated with overall diseases though *A* was recessive. The subgroup analysis based on ethnicity showed significant association between the *HIF1A* 1772 C/T polymorphism and overall disease for the Caucasian population under the all genetic models, which indicates that the *C* allele is associated with overall diseases. Again, the ethnicity subgroup showed a significant association between *HIF1A* 1790 G/A polymorphism and overall disease for the Asian population under recessive model only (*AA* vs. *AG* + *GG*), which indicates that the *A* allele is associated with overall diseases. The subgroup analysis based on disease type showed that *HIF1A* 1772 C/T is significantly associated with COPD, skin and diabetic complications diseases, where C is high risk factor for skin and diabetic complications (since, ORs > 1), but low risk factor for COPD (since, ORs < 1). This subgroup analysis results goes in favor of Ekberg et al. [65], and Bi et al. [66] for diabetic complications and Yu et al. [17] and Wei et al. [37] for COPD. The association of diabetic complication risk was also supported by a previous meta-analysis report [75]. Also the subgroup analysis results of *HIF1A* 1772 C/T polymorphism showed insignificant association with autoimmune diseases, inflammatory diseases, and preeclampsia under all five genetic models which goes in favor of Wipff et al. [31] and Feng et al. [25] for autoimmune disease, Torres et al. [27], Chachami et al. [32] and Senhaji et al. [41] for inflammatory disease, and Nava-Salazar et al. [34] for preeclampsia. The subgroup analysis results of *HIF1A* 1790 G/A polymorphism showed significant association with CVD under homozygote model (*AA* vs. *GG*) and diabetic complications under allelic (*A* vs. *G*) and homozygote (*AA* vs. *GG*) models. Also, the *HIF1A* 1790 G/A polymorphism showed significantly decreasing the risk of other (LDD, HAPC, Pressure injury) disease group under four genetic models (*A* vs. *G*, *AA* vs. *GG*, *AA* + *GA* vs. *GG*, *AA* vs. *GA* + *GG)* and insignificant association with inflammatory disease, COPD, autoimmune disease and preeclampsia under all genetic models. The association of diabetic complications contradicted the reports by Ren et al. [75]. The insignificant result of *HIF1A* 1790 G/A were supported by Bahadori et al. [26] for CVD, Torres et al. [27], Chachami et al. [32], Senhaji et al. [41], and Fernández-Torres et al. [20] for inflammatory disease, Putra et al. [39] for lung and Nava-Salazar et al. [34] for preeclampsia.

Thus the above discussion provided the significant evidence that the *HIF1A* gene is a risk factor for the development of COPD, CVD, skin disease and diabetic complications. Now it is required to explore the causality of HIF1A gene SNPs (1772 C/T and 1790 G/A) in the development of those disease by their expression analysis. Recently, some researchers studied single or multiple disease causing genes or SNPs by using network analysis or Mendelian randomization [76–82]. These SNPs can act as biological markers to locate the disease-causing genes that are regulated either directly or indirectly by those SNPs [83]. When SNPs occur within a gene or in a regulatory region near a gene, they are known as *cis*-acting factors, and they may play a more direct role in disease development by affecting the gene’s function. When SNPs occur far away from the disease causing genes, they are known as trans-acting factors. The *cis*- and *trans-*acting factors are usually considered as the causal and non-causal risk factors of disease development, respectively. SNPs can be silent due to its occurrence within the noncoding regions or may change the encoded amino acids due to its occurrence within the coding region. They may influence promoter or enhancer activities, messenger RNA (mRNA) stability, and subcellular localization of mRNAs and/or proteins and hence may develop disease. A post-transcriptional modification (PTM) in mRNA, known as N4-acetylcytidine (ac4C) that occurs on cytidine, plays a vital role in the stability and regulation of mRNA translation. There are at least 15 nucleotide modifications found in mRNA of which m6A and N1-methyladenosine (m1A) are similar in function to ac4C. They play a significant role in the translation process of mRNA and its stability that leads to the progression of several human diseases [84–87].

If SNPs (1772 C/T and 1790 G/A) of *HIF1A*gene data are available for COPD, CVD, skin disease and diabetic complications, and control samples, an effective disease prediction model may be developed by using a suitable machine learning technique including logistic classifier. For example, some recent studies developed SNPs based diseases prediction model [88, 89].

However, there were some limitations in this study, such as (i) the heterogeneity factors such as gender, age, smoking, drinking, blood pressure, family history, etc was not considered to estimate the combined effect for overall or subgroup analysis like as [56–60]. Because the dataset was generated through multiple diseases excluding cancer, so we cannot focus on specific behavior factor due to the insufficient information of GWAS studies. (ii) the metadata was collected considering the English language only, (iii) some subgroup analysis may be affected due to the small subgroup sample size and unavailable data due to limited GWAS studies.

In conclusion, this study made a consensus decision about the association of *HIF1A* gene polymorphisms with multiple diseases risks excluding cancers. The meta-analysis results showed that the *HIF1A* 1772 C/T polymorphism is not significantly associated with overall disease risks The *HIF1A* 1790 G/A polymorphism was associated with overall diseases under recessive model, where the allele *A* controls the diseases though it is recessive. The ethnicity subgroup analysis showed the significant association of *HIF1A* 1772 C/T polymorphism with overall disease for Caucasian population under all genetic models, where *C* allele controls the diseases, while *HIF1A* 1790 G/A polymorphism was significantly associated with overall disease for Asian population under a genetic model due to the influence of *A* allele. The subgroup analysis based on disease types showed that *HIF1A* 1772 C/T is significantly associated with chronic obstructive pulmonary disease (COPD), skin and diabetic complications diseases, where *C* allele is the high risk factor for skin and diabetic complications diseases, and low risk factor for COPD. The *HIF1A* 1790 G/A polymorphism showed significant association with CVD under homozygote model and diabetic complications under allelic and homozygote models. The rest of diseases showed insignificant association with *HIF1A* gene under all of five genetic models by the subgroup analysis. Taken together, the results of this study suggest that *HIF1A* could be a useful prognostic biomarker for COPD, CVD, skin disease and diabetic complication diseases. In future, availability of more SGA studies on the different ethnic populations might shed more lights to unveil and confirm the association of the *HIF1A* gene polymorphisms with different diseases.

## Supporting information

S1 Checklist PlosOne-meta-analysis-on-genetic-association-studies-checklist. (DOCX)

S2 Checklist PRISMA checklist. (DOCX)

S1 Data The dataset of *HIF1A* gene rs11549465 polymorphism. (XLSX)

S2 Data The dataset of *HIF1A* gene rs11549467 polymorphism. (XLSX)

S1 Table Quality assessment for included study in the meta-analysis. (DOCX)

S2 Table Heterogeneity analysis of *HIF1A* gene polymorphisms. (DOCX)

S3 Table Publication bias checking by using Egger’s linear regression and Begg’s test of *HIF1A* gene polymorphisms. (DOCX)

S4 Table Group of disease conducting this meta-analysis. (DOCX)

S1 File Forest plot of *HIF1A* gene polymorphisms (rs11549465, rs11549467) for four genetic models. (DOCX)

## References

[1] Iglesias P.; Penas C.; Barral-Cagiao L.; Pazos E.; Costoya J.A. A Bio-Inspired Hypoxia Sensor Using HIF1a-Oxygen-Dependent Degradation Domain. *Sci*. *Rep*. 2019, 9, doi: 10.1038/s41598-019-43618-4 31068630 PMC6506541

[2] Wenger R.H.; Stiehl D.P.; Camenisch G. Integration of Oxygen Signaling at the Consensus HRE. *Sci*. *STKE* 2005, 2005. doi: 10.1126/stke.3062005re12 16234508

[3] Semenza G.L. Oxygen-Dependent Regulation of Mitochondrial Respiration by Hypoxia-Inducible Factor 1. *Biochem*. *J*. 2007, 405. doi: 10.1042/BJ20070389 17555402

[4] CieSzczyk P.; Kalinski M.; Ostanek M.; Jascaniene N.; Krupecki K.; Ficek K.; et al. Variation in the Hif1a Gene in Elite Rowers. *J*. *Strength Cond*. *Res*. 2012, 26, doi: 10.1519/JSC.0b013e31824b876d 22476163

[5] Semenza G.L. Targeting HIF-1 for Cancer Therapy. *Nat*. *Rev*. *Cancer* 2003, 3. doi: 10.1038/nrc1187 13130303

[6] Pouysségur J.; Dayan F.; Mazure N.M. Hypoxia Signalling in Cancer and Approaches to Enforce Tumour Regression. *Nature* 2006, 441. doi: 10.1038/nature04400 16724055

[7] Jia X.; Hong Q.; Lei L.; Li D.; Li J.; Mo M.; et al. Basal and Therapy-Driven Hypoxia-Inducible Factor-1α Confers Resistance to Endocrine Therapy in Estrogen Receptor-Positive Breast Cancer. *Oncotarget* 2015, 6, doi: 10.18632/oncotarget.3257 25929338 PMC4496173

[8] Jiang Y.Z.; Liu Y.R.; Xu X.E.; Jin X.; Hu X.; Yu K.; et al. Transcriptome Analysis of Triple-Negative Breast Cancer Reveals an Integrated MRNA-LncRNA Signature with Predictive and Prognostic Value. *Cancer Res*. 2016, 76. doi: 10.1158/0008-5472.CAN-15-3284 26921339

[9] Clifford S.C.; Astuti D.; Hooper L.; Maxwell P.H.; Ratcliffe P.J.; Maher E.R. The PVHL-Associated SCF Ubiquitin Ligase Complex: Molecular Genetic Analysis of Elongin B and C, Rbx1 and HIF-1α in Renal Cell Carcinoma. *Oncogene* 2001, 20, doi: 10.1038/sj.onc.1204602 11526493

[10] Tanimoto K.; Yoshiga K.; Eguchi H.; Kaneyasu M.; Ukon K.; Kumazaki T.; et al. Hypoxia-Inducible Factor-1α Polymorphisms Associated with Enhanced Transactivation Capacity, Implying Clinical Significance. *Carcinogenesis* 2003, 24, doi: 10.1093/carcin/bgg132 12919954

[11] Yan Q.; Chen P.; Wang S.; Liu N.; Zhao P.; Gu A. Association between HIF-1α C1772T/G1790A Polymorphisms and Cancer Susceptibility: An Updated Systematic Review and Meta-Analysis Based on 40 Case-Control Studies. *BMC Cancer* 2014, 14, doi: 10.1186/1471-2407-14-950 25496056 PMC4301938

[12] Yang X.; Zhu H.C.; Zhang C.; Qin Q.; Liu J.; Xu L.P.; et al. HIF-1α 1772 C/T and 1790 G/A Polymorphisms Are Significantly Associated with Higher Cancer Risk: An Updated Meta-Analysis from 34 Case-Control Studies. *PLoS One* 2013, 8, doi: 10.1371/journal.pone.0080396 24260383 PMC3832403

[13] Wong B.W.; Marsch E.; Treps L.; Baes M.; Carmeliet P. Endothelial Cell Metabolism in Health and Disease: Impact of Hypoxia. *EMBO J*. 2017, 36, doi: 10.15252/embj.201696150 28637793 PMC5538796

[14] LaGory E.L.; Giaccia A.J. The Ever-Expanding Role of HIF in Tumour and Stromal Biology. *Nat*. *Cell Biol*. 2016, 18. doi: 10.1038/ncb3330 27027486 PMC4898054

[15] Anam M.T.; Ishika A.; Hossain M.B.; Jesmin A Meta-Analysis of Hypoxia Inducible Factor 1-Alpha (HIF1A) Gene Polymorphisms: Association with Cancers. *Biomark*. *Res*. 2015, 3, doi: 10.1186/s40364-015-0054-z 26715988 PMC4693423

[16] Harati-Sadegh M.; Kohan L.; Teimoori B.; Mehrabani M.; Salimi S. The Association of the Placental Hypoxia-Inducible Factor1-α Polymorphisms and HIF1-α MRNA Expression with Preeclampsia. *Placenta* 2018, 67, doi: 10.1016/j.placenta.2018.05.005 29941171

[17] Yu Z.G.; Wang B.Z.; Cheng Z.Z. The Association of Genetic Polymorphisms of Hypoxia Inducible Factor-1 Alpha and Vascular Endothelial Growth Factor with Increased Risk of Chronic Obstructive Pulmonary Disease: A Case–Control Study. *Kaohsiung J*. *Med*. *Sci*. 2017, 33, doi: 10.1016/j.kjms.2017.05.014 28865600 PMC11915911

[18] Hernández-Molina G.; Rodríguez-Pérez J.M.; Fernández-Torres J.; Lima G.; Pérez-Hernández N.; López-Reyes A.; et al. HIF1A (Rs11549465) and AKNA (Rs10817595) Gene Polymorphisms Are Associated with Primary Sjögren’s Syndrome. *Biomed Res*. *Int*. 2017, 2017, doi: 10.1155/2017/5845849 28484714 PMC5397622

[19] Vodolazkaia A.; Yesilyurt B.T.; Kyama C.M.; Bokor A.; Schols D.; Huskens D.; et al. Vascular Endothelial Growth Factor Pathway in Endometriosis: Genetic Variants and Plasma Biomarkers. *Fertil*. *Steril*. 2016, 105, doi: 10.1016/j.fertnstert.2015.12.016 26773192

[20] Fernández-Torres J.; Hernández-Díaz C.; Espinosa-Morales R.; Camacho-Galindo J.; Galindo-Sevilla N.D.C.; López-Macay Á.; et al. Polymorphic Variation of Hypoxia Inducible Factor-1 a (HIF1A) Gene Might Contribute to the Development of Knee Osteoarthritis: A Pilot Study. *BMC Musculoskelet*. *Disord*. 2015, 16, doi: 10.1186/s12891-015-0678-z 26293784 PMC4546180

[21] Hlatky M.A.; Quertermous T.; Boothroyd D.B.; Priest J.R.; Glassford A.J.; Myers R.M.; et al. Polymorphisms in Hypoxia Inducible Factor 1 and the Initial Clinical Presentation of Coronary Disease. *Am*. *Heart J*. 2007, 154, doi: 10.1016/j.ahj.2007.07.042 18035072

[22] Duran J.; Olavarría P.S.; Mola M.; Götzens V.; Carballo J.; Pelegrina E.M.; et al. Genetic Association Study of Coronary Collateral Circulation in Patients with Coronary Artery Disease Using 22 Single Nucleotide Polymorphisms Corresponding to 10 Genes Involved in Postischemic Neovascularization. *BMC Cardiovasc*. *Disord*. 2015, 15, doi: 10.1186/s12872-015-0027-z 25959001 PMC4493944

[23] Okur V.; Cetin O.; Cetin E.; Tepeli E.; Bulgu Y.; Yildirim C. HIF1A as a Major Vascular Endothelial Growth Factor Regulator: Do Its Polymorphisms Have an Association with Age-Related Macular Degeneration? *Clin*. *Exp*. *Ophthalmol*. 2015, 43, doi: 10.1111/ceo.12376 24995509

[24] Andraweera P.H.; Dekker G.A.; Thompson S.D.; Dissanayake V.H.W.; Jayasekara R.W.; Roberts C.T. Hypoxia-Inducible Factor-1α Gene Polymorphisms in Early and Late Onset Preeclampsia in Sinhalese Women. *Placenta* 2014, 35, doi: 10.1016/j.placenta.2014.04.008 24819156

[25] Feng C.C.; Ye Q.L.; Zhu Y.; Leng R.X.; Chen G.M.; Yang J.; et al. Lack of Association between the Polymorphisms of Hypoxia-Inducible Factor 1A (HIF1A) Gene and SLE Susceptibility in a Chinese Population. *Immunogenetics* 2014, 66, doi: 10.1007/s00251-013-0743-4 24232601

[26] Bahadori B.; Uitz E.; Mayer A.; Harauer J.; Dam K.; Truschnig-Wilders M.; et al. Polymorphisms of the Hypoxia-Inducible Factor 1 Gene and Peripheral Artery Disease. *Vasc*. *Med*. 2010, 15, doi: 10.1177/1358863X10379674 20926496

[27] Torres O.; Palomino-Morales R.; Vazquez-Rodriguez T.R.; Gamallo C.; Morado I.C.; Miranda-Filloy J.A.; et al. Lack of Association between Hypoxia Inducible Factor-1 Alpha Gene Polymorphisms and Biopsy-Proven Giant Cell Arteritis. *Clin*. *Exp*. *Rheumatol*. 2010, 28. 20412701

[28] Emanuele E.; Bertona M.; Geroldi D. A Multilocus Candidate Approach Identifies ACE and HIF1A as Susceptibility Genes for Cellulite. *J*. *Eur*. *Acad*. *Dermatology Venereol*. 2010, 24, doi: 10.1111/j.1468-3083.2009.03556.x 20059631

[29] Geza N.; Reka K.N.; Kereszturi E.; Somogyi A.; Szekely A.; Nemeth N.; et al. Association of Hypoxia Inducible Factor-1 Alpha Gene Polymorphism with Both Type 1 and Type 2 Diabetes in a Caucasian (Hungarian) Sample. *BMC Med*. *Genet*. 2009, 10, doi: 10.1186/1471-2350-10-79 19691832 PMC2736933

[30] Zheng Z.L.; Hwang Y.H.; Kim S.K.; Kim S.; Son M.J.; Ro H.; et al. Genetic Polymorphisms of Hypoxia-Inducible Factor-1 Alpha and Cardiovascular Disease in Hemodialysis Patients. *Nephron—Clin*. *Pract*. 2009, 113, doi: 10.1159/000228542 19602906

[31] Wipff J.; Dieude P.; Avouac J.; Tiev K.; Hachulla E.; Granel B.; et al. Association of Hypoxia-Inducible Factor 1A (HIF1A) Gene Polymorphisms with Systemic Sclerosis in a French European Caucasian Population. *Scand*. *J*. *Rheumatol*. 2009, 38, doi: 10.1080/03009740802629432 19306159

[32] Chachami G.; Kalousi A.; Papatheodorou L.; Lyberopoulou A.; Nasikas V.; Tanimoto K.; et al. An Association Study between Hypoxia Inducible Factor-1alpha (HIF-1α) Polymorphisms and Osteonecrosis. *PLoS One* 2013, 8, doi: 10.1371/journal.pone.0079647 24260273 PMC3832621

[33] Lin W.P.; Wang X.J.; Wang C.R.; Zhang L.Q.; Li N.; Wang F.S.; et al. Polymorphism in the Hypoxia-Inducible Factor 1alpha Gene May Confer Susceptibility to LDD in Chinese Cohort. *PLoS One* 2013, 8, doi: 10.1371/journal.pone.0073158 23991178 PMC3753262

[34] Nava-Salazar S.; Snchez-Rodríguez E.N.; Mendoza-Rodríguez C.A.; Moran C.; Romero-Arauz J.F.; Cerbán M.A. Polymorphisms in the Hypoxia-Inducible Factor 1 Alpha Gene in Mexican Patients with Preeclampsia: A Case-Control Study. *BMC Res*. *Notes* 2011, 4, doi: 10.1186/1756-0500-4-68 21414224 PMC3076269

[35] Yamada N.; Horikawa Y.; Oda N.; Iizuka K.; Shihara N.; Kishi S.; et al. Genetic Variation in the Hypoxia-Inducible Factor-1α Gene Is Associated with Type 2 Diabetes in Japanese. *J*. *Clin*. *Endocrinol*. *Metab*. 2005, 90, doi: 10.1210/jc.2005-0991 16046581

[36] Chen Y.; Jiang C.; Luo Y.; Liu F.; Gao Y. Interaction of CARD14, SENP1 and VEGFA Polymorphisms on Susceptibility to High Altitude Polycythemia in the Han Chinese Population at the Qinghai-Tibetan Plateau. *Blood Cells*, *Mol*. *Dis*. 2016, 57, doi: 10.1016/j.bcmd.2015.11.005 26852650

[37] Wei W.T.; Li B.; Chen M.; Jia H.R.; Zhang H.X. Associations between HIF-1α Polymorphisms C1772T and G1790A and Susceptibility to Chronic Obstructive Pulmonary Disease. *Genet*. *Mol*. *Res*. 2015, 14, doi: 10.4238/2015.December.21.2 26782374

[38] de Carvalho Fraga C.A.; Alves L.R.; Marques-Silva L.; de Sousa A.A.; Jorge A.S.B.; de Jesus S.F.; et al. High HIF-1α Expression Genotypes in Oral Lichen Planus. *Clin*. *Oral Investig*. 2013, 17, doi: 10.1007/s00784-013-0920-8 23334242

[39] Putra A.C.; Tanimoto K.; Arifin M.; Antariksa B.; Hiyama K. Genetic Variations in Detoxification Enzymes and HIF-1α in Japanese Patients with COPD. *Clin*. *Respir*. *J*. 2013, 7, doi: 10.1111/j.1752-699X.2011.00255.x 21651746

[40] Liu Q.; Liang Y.; Zou P.; Ni W.X.; Li Y.G.; Chen S.M. Hypoxia-Inducible Factor-1α Polymorphisms Link to Coronary Artery Collateral Development and Clinical Presentation of Coronary Artery Disease. *Biomed*. *Pap*. 2013, 157, doi: 10.5507/bp.2013.061 24089028

[41] Senhaji N.; Nadifi S.; Zaid Y.; Serrano A.; Leon Rodriguez D.A.; Serbati N.; et al. Polymorphisms in Oxidative Pathway Related Genes and Susceptibility to Inflammatory Bowel Disease. *World J*. *Gastroenterol*. 2017, 23, doi: 10.3748/wjg.v23.i47.8300 29307990 PMC5743501

[42] Zafar U.; Ali Z.; Khaliq S.; Lone K. Association between Hypoxia-Inducible Factor-1 Alpha Rs11549465 (1772 C>T) Polymorphism and Metabolic Syndrome. *J*. *Pak*. *Med*. *Assoc*. 2021, 71, 1832–1837, doi: 10.47391/JPMA.03-434 34410257

[43] Sheng Z.L.; Ju C.W.; Yan G.L.; Chen Z.P.; Pan X.D.; Lu W.B.; et al. The Relevance of HIF1A Gene Polymorphisms and Primary Hypertensive Left Ventricular Hypertrophy in Chinese Han Population. *Eur*. *Rev*. *Med*. *Pharmacol*. *Sci*. 2019, 23, 8095–8100, doi: 10.26355/eurrev_201909_19027 31599436

[44] Urganci B.; Acikbas I.; Rezzan Er F. Investigation of Immunovascular Polymorphisms and Intersections in Psoriasis. *Indian J*. *Dermatol*. 2019, 64, doi: 10.4103/ijd.IJD_422_18 31148856 PMC6537683

[45] Liu Y.-H.; Guo C.; Sun Y.-Q.; Li Q. Polymorphisms in HIF-1a Gene Are Not Associated with Diabetic Retinopathy in China. *World J*. *Diabetes* 2021, 12, 1304–1311, doi: 10.4239/wjd.v12.i8.1304 34512895 PMC8394233

[46] Takagi K.; Kawamoto M.; Higuchi T.; Tochimoto A.; Harigai M.; Kawaguchi Y. Single Nucleotide Polymorphisms of the HIF1A Gene Are Associated with Susceptibility to Pulmonary Arterial Hypertension in Systemic Sclerosis and Contribute to SSc-PAH Disease Severity. *Int*. *J*. *Rheum*. *Dis*. 2020, 23, 674–680, doi: 10.1111/1756-185X.13822 32144871

[47] Saravani M.; Rokni M.; Mehrbani M.; Amirkhosravi A.; Faramarz S.; Fatemi I.; et al. The Evaluation of VEGF and HIF-1α Gene Polymorphisms and Multiple Sclerosis Susceptibility. *J*. *Gene Med*. 2019, 21, 1–7, doi: 10.1002/jgm.3132 31652374

[48] Qin L.; Shu L.; Zhong J.; Pan H.; Guo J.; Sun Q.; et al. Association of HIF1A and Parkinson’s Disease in a Han Chinese Population Demonstrated by Molecular Inversion Probe Analysis. *Neurol*. *Sci*. 2019, 40, 1927–1931, doi: 10.1007/s10072-019-03905-4 31025220

[49] Tsukatani T.; Minematsu T.; Dai M.; Tamai N.; Nakagami G.; Sugama J.; et al. Polymorphism Analysis of Candidate Risk Genes for Pressure Injuries in Older Japanese Patients: A Cross-Sectional Study at a Long-Term Care Hospital. *Wound Repair Regen*. 2021, 29, 741–751, doi: 10.1111/wrr.12912 33819344

[50] Pichu S.; Sathiyamoorthy J.; Krishnamoorthy E.; Umapathy D.; Viswanathan V. Impact of the Hypoxia Inducible Factor-1α (HIF-1α) Pro582ser Polymorphism and Its Gene Expression on Diabetic Foot Ulcers. *Diabetes Res*. *Clin*. *Pract*. 2015, 109, doi: 10.1016/j.diabres.2015.05.014 26113285

[51] Ekberg N.R.; Eliasson S.; Li Y.W.; Zheng X.; Chatzidionysiou K.; Falhammar H.; et al. Protective Effect of the HIF-1A Pro582Ser Polymorphism on Severe Diabetic Retinopathy. *J*. *Diabetes Res*. 2019, 2019, doi: 10.1155/2019/2936962 31214621 PMC6535890

[52] Bi Y.X.; Yu L.; Jin G.X. Correlation between Polymorphisms of Hypoxia-Inducible Factor-1α Pro582Ser and Type 2 Diabetic Nephropathy. *Genet*. *Mol*. *Res*. 2015, 14, 14503–14509, doi: 10.4238/2015.November.18.13 26600509

[53] Gu H.F.; Zheng X.; Seman N.A.; Gu T.; Botusan I.R.; Sunkari V.G.; et al. Impact of the Hypoxia-Inducible Factor-1 α (HIF1A) Pro582Ser Polymorphism on Diabetes Nephropathy. *Diabetes Care* 2013, 36, 415–421, doi: 10.2337/dc12-1125 22991450 PMC3554309

[54] Pichu S.; Vimalraj S.; Sathiyamoorthy J.; Viswanathan V. Association of Hypoxia Inducible Factor-1 Alpha Exon 12 Mutation in Diabetic Patients with and without Diabetic Foot Ulcer. *Int*. *J*. *Biol*. *Macromol*. 2018, 119, 833–837, doi: 10.1016/j.ijbiomac.2018.08.011 30086330

[55] Harun-Or-Roshid M.; Ali M.B.; Jesmin; Mollah M.N.H. Statistical Meta-Analysis to Investigate the Association between the Interleukin-6 (IL-6) Gene Polymorphisms and Cancer Risk. *PLoS One* 2021, 16, 1–26, doi: 10.1371/journal.pone.0247055 33684135 PMC7939379

[56] Xu M.Q.; Ye Z.; Hu F.B.; He L. Quantitative Assessment of the Effect of Angiotensinogen Gene Polymorphisms on the Risk of Coronary Heart Disease. *Circulation* 2007, 116, 1356–1366, doi: 10.1161/CIRCULATIONAHA.107.728857 17846284

[57] Xu M.; Sham P.; Ye Z.; Lindpaintner K.; He L. A1166C Genetic Variation of the Angiotensin II Type I Receptor Gene and Susceptibility to Coronary Heart Disease: Collaborative of 53 Studies with 20,435 Cases and 23,674 Controls. *Atherosclerosis* 2010, 213, 191–199, doi: 10.1016/j.atherosclerosis.2010.07.046 20732682

[58] Xu M.; Lin Z. Genetic Influences of Dopamine Transport Gene on Alcohol Dependence: A Pooled Analysis of 13 Studies with 2483 Cases and 1753 Controls. *Prog*. *Neuro-Psychopharmacology Biol*. *Psychiatry* 2011, 35, doi: 10.1016/j.pnpbp.2010.11.001 21078357 PMC5335908

[59] Wu Y.; Cao H.; Baranova A.; Huang H.; Li S.; Cai L.; et al. Multi-Trait Analysis for Genome-Wide Association Study of Five Psychiatric Disorders. *Transl*. *Psychiatry* 2020, 10, doi: 10.1038/s41398-020-00902-6 32606422 PMC7326916

[60] Jiang L.; Wang K.; Lo K.; Zhong Y.; Yang A.; Fang X.; et al. Sex-Specific Association of Circulating Ferritin Level and Risk of Type 2 Diabetes: A Dose-Response Meta-Analysis of Prospective Studies. *J*. *Clin*. *Endocrinol*. *Metab*. 2019, 104, 4539–4551, doi: 10.1210/jc.2019-00495 31074789

[61] Liberati A.; Altman D.G.; Tetzlaff J.; Mulrow C.; Gøtzsche P.C.; Ioannidis J.P.A.; et al. The PRISMA Statement for Reporting Systematic Reviews and Meta-Analyses of Studies That Evaluate Health Care Interventions: Explanation and Elaboration. *Ann*. *Intern*. *Med*. 2009, 151. 10.7326/0003-4819-151-4-200908180-00136 19622512

[62] Stang A. Critical Evaluation of the Newcastle-Ottawa Scale for the Assessment of the Quality of Nonrandomized Studies in Meta-Analyses. *Eur*. *J*. *Epidemiol*. 2010, 25. doi: 10.1007/s10654-010-9491-z 20652370

[63] Liu I.-M.; Agresti A. Mantel-Haenszel-Type Inference for Cumulative Odds Ratios with a Stratified Ordinal Response. *Biometrics* 1996, 52, doi: 10.2307/2532838 8962452

[64] Ravi S. Book Review: Methods for Meta-Analysis in Medical Research. *Stat*. *Methods Med*. *Res*. 2005, 14, doi: 10.1191/0962280205sm401xx

[65] Cochran W.G. The Combination of Estimates from Different Experiments. *Biometrics* 1954, 10, doi: 10.2307/3001666

[66] Higgins J.P.T.; Thompson S.G. Quantifying Heterogeneity in a Meta-Analysis. *Stat*. *Med*. 2002, 21, doi: 10.1002/sim.1186 12111919

[67] Valenzuela C. 2 Solutions for Estimating Odds Ratios with Zeros. *Rev*. *Med*. *Chil*. 1993, 121. 8085071

[68] Egger M.; Smith G.D.; Schneider M.; Minder C. Bias in Meta-Analysis Detected by a Simple, Graphical Test. *Br*. *Med*. *J*. 1997, 315, doi: 10.1136/bmj.315.7109.629 9310563 PMC2127453

[69] Begg C.B.; Mazumdar M. Operating Characteristics of a Rank Correlation Test for Publication Bias. *Biometrics* 1994, 50, doi: 10.2307/2533446 7786990

[70] Wacholder S.; Chanock S.; Garcia-Closas M.; El Ghormli L.; Rothman N. Assessing the Probability That a Positive Report Is False: An Approach for Molecular Epidemiology Studies. *J*. *Natl*. *Cancer Inst*. 2004, 96, 434–442, doi: 10.1093/jnci/djh075 15026468 PMC7713993

[71] Zhou L.; Zheng Y.; Tian T.; Liu K.; Wang M.; Lin S.; et al. Associations of Interleukin-6 Gene Polymorphisms with Cancer Risk: Evidence Based on 49,408 Cancer Cases and 61,790 Controls. *Gene* 2018, 670, 136–147, doi: 10.1016/j.gene.2018.05.104 29842912

[72] Marcus P.M.; Vineis P.; Rothman N. NAT2 Slow Acetylation and Bladder Cancer Risk: A Meta-Analysis of 22 Case-Control Studies Conducted in the General Population. *Pharmacogenetics* 2000, 10, doi: 10.1097/00008571-200003000-00003 10761999

[73] Engel L.S.; Taioli E.; Pfeiffer R.; Garcia-Closas M.; Marcus P.M.; Lan Q.; et al. Pooled Analysis and Meta-Analysis of Glutathione S-Transferase M1 and Bladder Cancer: A HuGE Review. *Am*. *J*. *Epidemiol*. 2002, 156. doi: 10.1093/aje/kwf018 12117698

[74] He J.; Zou Y.; Liu X.; Zhu J.; Zhang J.; Zhang R.; et al. Association of Common Genetic Variants in Pre-MicroRNAs and Neuroblastoma Susceptibility: A Two-Center Study in Chinese Children. *Mol*. *Ther*.*—Nucleic Acids* 2018, 11, doi: 10.1016/j.omtn.2018.01.003 29858046 PMC5849804

[75] Ren H.; Luo J.Q.; Gao Y.C.; Chen M.Y.; Chen X.P.; Zhou H.H.; et al. Genetic Association of Hypoxia Inducible Factor 1-Alpha (HIF1A) Pro582Ser Polymorphism with Risk of Diabetes and Diabetic Complications. *Aging (Albany*. *NY)*. 2020, 12, 12783–12798, doi: 10.18632/aging.103213 32658866 PMC7377833

[76] Zhang F.; Baranova A.; Zhou C.; Cao H.; Chen J.; Zhang X.; et al. Causal Influences of Neuroticism on Mental Health and Cardiovascular Disease. *Hum*. *Genet*. 2021, 140, doi: 10.1007/s00439-021-02288-x 33973063

[77] Zhang F.; Rao S.; Cao H.; Zhang X.; Wang Q.; Xu Y.; et al. Genetic Evidence Suggests Posttraumatic Stress Disorder as a Subtype of Major Depressive Disorder. *J*. *Clin*. *Invest*. 2021, doi: 10.1172/jci145942 33905376 PMC8803333

[78] Wang X.; Fang X.; Zheng W.; Zhou J.; Song Z.; Xu M.; et al. Genetic Support of A Causal Relationship Between Iron Status and Type 2 Diabetes: A Mendelian Randomization Study. *J*. *Clin*. *Endocrinol*. *Metab*. 2021, 106, doi: 10.1210/clinem/dgab454 34147035 PMC8530720

[79] Hou L.; Xu M.; Yu Y.; Sun X.; Liu X.; Liu L.; et al. Exploring the Causal Pathway from Ischemic Stroke to Atrial Fibrillation: A Network Mendelian Randomization Study. *Mol*. *Med*. 2020, 26, doi: 10.1186/s10020-019-0133-y 31941463 PMC6964084

[80] Zhang F.; Baranova A. Smoking Quantitatively Increases Risk for COVID-19. *Eur*. *Respir*. *J*. 2021, doi: 10.1183/13993003.01273-2021 34326191 PMC8340618

[81] Hu P.; Jiao R.; Jin L.; Xiong M. Application of Causal Inference to Genomic Analysis: Advances in Methodology. *Front*. *Genet*. 2018, 9. doi: 10.3389/fgene.2018.00238 30042787 PMC6048229

[82] Kou N.; Zhou W.; He Y.; Ying X.; Chai S.; Fei T.; et al. A Mendelian Randomization Analysis to Expose the Causal Effect of IL-18 on Osteoporosis Based on Genome-Wide Association Study Data. *Front*. *Bioeng*. *Biotechnol*. 2020, 8, doi: 10.3389/fbioe.2020.00201 32266232 PMC7099043

[83] Gray I.C.; Campbell D.A.; Spurr N.K. Single Nucleotide Polymorphisms as Tools in Human Genetics. *Hum*. *Mol*. *Genet*. 2000, 9. doi: 10.1093/hmg/9.16.2403 11005795

[84] Zhou X.; Li Q.; Xu J.; Zhang X.; Zhang H.; Xiang Y.; et al. The Aberrantly Expressed MiR-193b-3p Contributes to Preeclampsia through Regulating Transforming Growth Factor-β Signaling. *Sci*. *Rep*. 2016, 6, doi: 10.1038/srep19910 26822621 PMC4731805

[85] Yan X.; Zhao X.; Li J.; He L.; Xu M. Effects of Early-Life Malnutrition on Neurodevelopment and Neuropsychiatric Disorders and the Potential Mechanisms. *Prog*. *Neuro-Psychopharmacology Biol*. *Psychiatry* 2018, 83. doi: 10.1016/j.pnpbp.2017.12.016 29287829

[86] Jin G.; Xu M.; Zou M.; Duan S. The Processing, Gene Regulation, Biological Functions, and Clinical Relevance of N4-Acetylcytidine on RNA: A Systematic Review. *Mol*. *Ther*.*—Nucleic Acids* 2020, 20. 10.1016/j.omtn.2020.01.037 PMC7068197 32171170

[87] Zheng S.; Zhao T.; Yuan S.; Yang L.; Ding J.; Cui L.; et al. Immunodeficiency Promotes Adaptive Alterations of Host Gut Microbiome: An Observational Metagenomic Study in Mice. *Front*. *Microbiol*. 2019, 10, doi: 10.3389/fmicb.2019.02415 31781050 PMC6853035

[88] Liu M.; Li F.; Yan H.; Wang K.; Ma Y.; Shen L.; et al. A Multi-Model Deep Convolutional Neural Network for Automatic Hippocampus Segmentation and Classification in Alzheimer’s Disease. *Neuroimage* 2020, 208, doi: 10.1016/j.neuroimage.2019.116459 31837471

[89] Yu H.; Pan R.; Qi Y.; Zheng Z.; Li J.; Li H.; et al. LEPR Hypomethylation Was Significantly Associated with Gastric Cancer in Males. *Exp*. *Mol*. *Pathol*. 2020, 116, doi: 10.1016/j.yexmp.2020.104493 32659237

