# Supplementary material for: Association of hypoxia inducible factor 1-Alpha gene polymorphisms with multiple disease risks: A comprehensive meta-analysis
Source: PLoS One. 2022 Aug 16;17(8):e0273042. doi: 10.1371/journal.pone.0273042 (PMC9380912; doi:10.1371/journal.pone.0273042)
Supplement: S2 Checklist — (DOCX) [file pone.0273042.s002.docx]

**Meta-analysis on Genetic Association Studies Checklist | PLOS ONE**

|  | Item | Section name and paragraph number within manuscript |
| --- | --- | --- |
|  | **Introduction** |  |
| 1 | Provide a detailed justification for the polymorphism studied; if a single polymorphism was analyzed, give details as to why others were not included in the meta-analysis. | Section: Introduction,  Paragraph: 1^st^ |
| 2 | Provide a detailed justification for the population(s) and clinical condition studied. | Section: Introduction,  Paragraph: 1^st^  and 2^nd^ |
|  | **Methods** |  |
| 3 | Provide full details of the search strategy employed; outline the full electronic search strategy –specific combination of keywords and any limits applied- for at least one database. Specify whether synonyms of polymorphisms/genes (e.g. SNP number) were searched. | Section: Materials and methods,  Sub-section: Search strategy,  Paragraph: 1^st^ |
| 4 | Report full details on the inclusion and exclusion criteria applied for selecting studies.  *Please list the excluded articles and the reasons for exclusion of each article in a supplementary file.* | Section: Materials and methods,  Sub-section: Eligibility criteria,  Paragraph: 1^st^ |
| 5 | Provide details on how the quality of the studies included in the analyses was assessed. | Section: Materials and methods,  Sub-section: Data extraction,  Paragraph: 1st |
| 6 | Describe steps taken to contact study authors to identify additional studies and to request missing data. | Section: Materials and methods (Search strategy, Eligibility criteria, Data extraction); |
| 7 | Describe how environmental effects were adjusted for, if this adjustment was not conducted, outline the reasons for this. | Section: Discussion and conclusion (4^th^ paragraph- Limitations) |
| 8 | Describe the methods of handling heterogeneity/between-study variance. | Section: Materials and methods,  Sub-section: Statistical meta-analysis (Supplimentary file (S1_Table.docx) |
| 9 | Describe how the Hardy-Weinberg equilibrium and linkage disequilibrium were assessed. | Section: Materials and methods,  Sub-section: Data extraction and statistical meta-analysis |
| 10 | Describe and justify the choice of model for the analyses (per-allele vs per-genotype vs genetic model-free, random effects vs fixed effects). | Section: Materials and methods,  Sub-section: Statistical meta-analysis |
| 11 | Describe whether a sensitivity analysis has been completed. | Section: Materials and methods, Results,  Sub-section: Statistical meta-analysis, Sensitivity analysis, respectively |
| 12 | Describe whether an assessment of the effects of population stratification has been conducted. | Section: Materials and methods,  Sub-section: Data extraction, Table 1. |
| 13 | Describe whether study-specific results have been assessed and if so the reasons for this (e.g. forest plot). | Section: Results and discussion  (Figure 2, 3, S1-S8) |
|  | **Results** |  |
| 14 | Include flow diagram for the studies included in the meta-analysis as the first figure for the manuscript | Section: Results and discussion,  Sub-section: Study characteristics,  Paragraph: 1^st^ and Figure 1. |
| 15 | Report details on allele/genotype prevalence. | Section: Results and discussion,  Sub-section: Quantitative synthesis (HIF1A 1772 C/T, HIF1A 1790 G/A), Table 2, 3. |
| 16 | Report the effect size estimates and p values for each analysis. | Section: Results and discussion ,  Sub-section: Quantitative synthesis (HIF1A 1772 C/T, HIF1A 1790 G/A), Table 2, 3. |
|  | **Discussion** |  |
| 17 | Discuss the limitations of the meta-analysis, including genotyping errors/bias and publication bias. | Section: Discussion and conclusion (4th paragraph- Limitations) , Publication bias checking |
| 18 | If the meta-analysis identifies an association within a subgroup of the population studied but not another, discuss the implications of these results, and if applicable the possibility of subgroup-specific publication bias. | Section: Discussion and conclusion, Publication bias checking |
| 19 | Discuss the suitability of the sample size employed to the research question and the power of the study. | Section: Discussion and conclusion,  Sub-section: Study characteristics (1^st^ paragraph), False positive report probability (FPRP) and power analyses (1^st^ paragraph) |
