# Supplementary material for: Association of hypoxia inducible factor 1-Alpha gene polymorphisms with multiple disease risks: A comprehensive meta-analysis
Source: PLoS One. 2022 Aug 16;17(8):e0273042. doi: 10.1371/journal.pone.0273042 (PMC9380912; doi:10.1371/journal.pone.0273042)
Supplement: S1 Table — (DOCX) [file pone.0273042.s005.docx]

**Table S1.** Quality assessment for included study in the meta-analysis

| **Study ID** | **Selection** | | | | **Comparability** | **Exposure** | | | | **Total scores** |
| --- | --- | --- | --- | --- | --- | --- | --- | --- | --- | --- |
|  | **Adequate definition of cases** | **Representativeness of cases** | **Selection of control subjects** | **Definition of control subjects** | **Comparability of cases and controls on the basis of the design or analysis** | **Exposure assessment** | **Same method of ascertainment for all subjects** | **Nonresponse rate** | |  |
| **rs11549465** | | | | | | | | | | |
| Harati-Sadegh et al. [16] | ★ | ★ | ★ | ★ | ★ ☆ | ★ | ★ | ★ | 8 | |
| Yu et al. [17] | ★ | ★ | ☆ | ★ | ★ ★ | ★ | ★ | ★ | 8 | |
| Hernandez-Molina et al. [28] | ★ | ★ | ☆ | ★ | ☆ ☆ | ★ | ★ | ★ | 6 | |
| Fernandez-Torres et al. [49] | ★ | ★ | ☆ | ★ | ★ ☆ | ★ | ★ | ★ | 7 | |
| Hlatky et al. [50] | ★ | ☆ | ☆ | ★ | ★ ☆ | ★ | ★ | ★ | 6 | |
| Duran et al. [51] | ★ | ★ | ☆ | ☆ | ★ ★ | ★ | ★ | ★ | 7 | |
| Okur et al. [52] | ★ | ★ | ☆ | ★ | ☆ ★ | ★ | ★ | ★ | 7 | |
| Andraweera et al. [53] | ★ | ★ | ☆ | ★ | ★ ★ | ★ | ★ | ★ | 8 | |
| Feng et al. [54] | ★ | ★ | ★ | ★ | ★ ★ | ★ | ★ | ★ | 9 | |
| Torres et al. [19] | ★ | ★ | ☆ | ★ | ★ ★ | ★ | ★ | ★ | 8 | |
| Emanuele et al. [20] | ★ | ★ | ☆ | ★ | ★ ☆ | ★ | ★ | ★ | 7 | |
| Geza et al. (a) [21] | ★ | ★ | ☆ | ★ | ★ ★ | ★ | ★ | ★ | 8 | |
| Geza et al. (b) [21] | ★ | ★ | ☆ | ★ | ★ ★ | ★ | ★ | ★ | 8 | |
| Zheng et al. [22] | ★ | ★ | ★ | ★ | ★ ★ | ★ | ★ | ★ | 9 | |
| Wipff et al. [23] | ★ | ★ | ☆ | ★ | ★ ★ | ★ | ★ | ★ | 8 | |
| Chachami et al. [24] | ★ | ★ | ★ | ★ | ★ ★ | ★ | ★ | ★ | 9 | |
| Lin et al. [25] | ★ | ★ | ☆ | ★ | ★ ★ | ★ | ★ | ★ | 8 | |
| Nava-Salazar et al. [26] | ★ | ★ | ☆ | ★ | ★ ☆ | ★ | ★ | ★ | 7 | |
| Yamada et al. [27] | ★ | ★ | ★ | ★ | ★ ★ | ★ | ★ | ★ | 9 | |
| Chen et al. [29] | ★ | ★ | ★ | ★ | ★ ☆ | ★ | ★ | ★ | 8 | |
| Wei et al. [30] | ★ | ★ | ☆ | ★ | ★ ★ | ★ | ★ | ★ | 8 | |
| de Carvalho Fraga et al. [31] | ★ | ★ | ★ | ★ | ★ ☆ | ★ | ★ | ★ | 8 | |
| Putra et al. [32] | ★ | ★ | ★ | ★ | ☆ ☆ | ★ | ★ | ★ | 7 | |
| Q. Liu et al. [33] | ★ | ★ | ★ | ★ | ☆ ☆ | ★ | ★ | ★ | 7 | |
| Zafar et al. [35] | ★ | ★ | ★ | ★ | ★ ☆ | ★ | ★ | ★ | 8 | |
| Sheng et al. [36] | ★ | ★ | ☆ | ★ | ★ ★ | ★ | ★ | ★ | 8 | |
| Urganci et al. [37] | ★ | ★ | ☆ | ★ | ☆ ★ | ★ | ★ | ★ | 7 | |
| Liu et al. (a) [38] | ★ | ★ | ☆ | ★ | ★ ★ | ★ | ★ | ★ | 8 | |
| Liu et al. (b) [38] | ★ | ★ | ☆ | ★ | ★ ★ | ★ | ★ | ★ | 8 | |
| Takagi et al. [40] | ★ | ★ | ☆ | ★ | ★ ★ | ★ | ☆ | ★ | 7 | |
| Saravani et al. [41] | ★ | ★ | ☆ | ★ | ★ ★ | ★ | ★ | ★ | 8 | |
| Qin et al. [42] | ★ | ★ | ★ | ★ | ★ ★ | ★ | ★ | ★ | 9 | |
| Tsukatani et al. [43] | ★ | ★ | ★ | ★ | ★ ★ | ★ | ★ | ★ | 9 | |
| Pichu et al. (a) [44] | ★ | ★ | ☆ | ★ | ★ ☆ | ★ | ★ | ★ | 7 | |
| Pichu et al. (b) [44] | ★ | ★ | ☆ | ★ | ★ ☆ | ★ | ★ | ★ | 7 | |
| Ekberg et al. [45] | ★ | ★ | ☆ | ☆ | ☆ ★ | ★ | ★ | ★ | 6 | |
| Bi et al. [46] | ★ | ★ | ☆ | ☆ | ★ ★ | ★ | ★ | ★ | 7 | |
| Gu et al. [47] | ★ | ★ | ☆ | ☆ | ★ ★ | ★ | ★ | ★ | 7 | |
| **rs11549467** | | | | | | | | | | |
| Harati-Sadegh et al. [16] | ★ | ★ | ★ | ★ | ★ ☆ | ★ | ★ | ★ | 8 | |
| Senhaji et al. [34] | ★ | ★ | ☆ | ★ | ★ ★ | ★ | ☆ | ★ | 7 | |
| Yu et al. [17] | ★ | ★ | ☆ | ★ | ★ ★ | ★ | ★ | ★ | 8 | |
| Hernandez-Molina et al. [28] | ★ | ★ | ☆ | ★ | ☆ ☆ | ★ | ★ | ★ | 6 | |
| Fernandez-Torres et al. [49] | ★ | ★ | ☆ | ★ | ☆ ★ | ★ | ★ | ★ | 7 | |
| Hlatky et al. [50] | ★ | ★ | ☆ | ★ | ★ ☆ | ★ | ★ | ★ | 7 | |
| Bahadori et al. [18] | ★ | ★ | ★ | ★ | ☆ ☆ | ★ | ★ | ★ | 7 | |
| Torres et al. [19] | ★ | ★ | ☆ | ★ | ☆ ★ | ★ | ★ | ★ | 7 | |
| Chachami et al. [24] | ★ | ★ | ★ | ★ | ★ ★ | ★ | ★ | ★ | 9 | |
| Lin et al. [25] | ★ | ★ | ☆ | ★ | ★ ★ | ★ | ★ | ★ | 8 | |
| Nava-Salazar et al. [26] | ★ | ★ | ☆ | ★ | ★ ☆ | ★ | ★ | ★ | 7 | |
| Yamada et al. [27] | ★ | ★ | ★ | ★ | ★ ★ | ★ | ★ | ★ | 9 | |
| Chen et al. [29] | ★ | ★ | ★ | ★ | ★ ☆ | ★ | ★ | ★ | 8 | |
| Wei et al. [30] | ★ | ★ | ☆ | ★ | ★ ★ | ★ | ★ | ★ | 8 | |
| Putra et al. [32] | ★ | ★ | ★ | ★ | ☆ ☆ | ★ | ★ | ★ | 7 | |
| Q. Liu et al. [33] | ★ | ★ | ★ | ★ | ☆ ☆ | ★ | ★ | ★ | 7 | |
| Sheng et al. [36] | ★ | ★ | ☆ | ★ | ★ ★ | ★ | ★ | ★ | 8 | |
| Liu et al. (a) [38] | ★ | ★ | ☆ | ★ | ★ ★ | ★ | ★ | ★ | 8 | |
| Liu et al. (b) [38] | ★ | ★ | ☆ | ★ | ★ ★ | ★ | ★ | ★ | 8 | |
| Takagi et al. [40] | ★ | ★ | ☆ | ★ | ★ ★ | ★ | ☆ | ★ | 7 | |
| Qin et al. [42] | ★ | ★ | ★ | ★ | ★ ★ | ★ | ★ | ★ | 9 | |
| Tsukatani et al. [43] | ★ | ★ | ★ | ★ | ★ ★ | ★ | ★ | ★ | 9 | |
| Pichu et al. (a) [48] | ★ | ★ | ★ | ★ | ★ ☆ | ★ | ★ | ★ | 8 | |
| Pichu et al. (b) [48] | ★ | ★ | ★ | ★ | ★ ☆ | ★ | ★ | ★ | 8 | |

★ (black star ) represents one score given; ☆ (hollowed star) represents one score not given;
