# Supplementary material for: Association of hypoxia inducible factor 1-Alpha gene polymorphisms with multiple disease risks: A comprehensive meta-analysis
Source: PLoS One. 2022 Aug 16;17(8):e0273042. doi: 10.1371/journal.pone.0273042 (PMC9380912; doi:10.1371/journal.pone.0273042)
Supplement: S2 Table — (DOCX) [file pone.0273042.s006.docx]

**Table S1.** Heterogeneity analysis of the HIF1A 1722 T/C and 1790 G/A polymorphisms

| **Types of Diseases** | **Genetic Model** | **Study number** | **Model** | **OR (95% CI)** | **Z-val** | **p-value** | **Q** | **d.f** | **p-val.Q** | **Tau2** | **I2 %** |
| --- | --- | --- | --- | --- | --- | --- | --- | --- | --- | --- | --- |
| **rs11549465 (1722 T/C)** | | | | | | | | | | | |
| Overall | C vs. T | 38 | REM | 1.12 [0.97; 1.29] | 1.59 | 0.113 | 119.65 | 37 | 0.000 | 0.120 | 69.1 |
|  | CC vs. TT | 38 | FEM | 1.16 [0.94; 1.44] | 1.42 | 0.154 | 34.17 | 37 | 0.602 | 0.100 | 0.0 |
|  | CT vs. TT | 38 | REM | 1.15 [0.83; 1.59] | 0.85 | 0.395 | 55.55 | 37 | 0.026 | 0.330 | 33.4 |
|  | CC + CT vs. TT | 38 | REM | 1.14 [0.86; 1.51] | 0.89 | 0.375 | 48.97 | 37 | 0.090 | 0.210 | 24.4 |
|  | CC vs. CT + TT | 38 | REM | 1.1 [0.93; 1.31] | 1.13 | 0.257 | 128.09 | 37 | 0.000 | 0.170 | 71.1 |
| Preeclampsia | C vs. T | 3 | REM | 1.03 [0.62; 1.7] | 0.11 | 0.911 | 5.2 | 2 | 0.074 | 0.110 | 61.6 |
|  | CC vs. TT | 3 | FEM | 0.7 [0.26; 1.91] | -0.7 | 0.483 | 1.46 | 2 | 0.482 | 0.100 | 0.0 |
|  | CT vs. TT | 3 | FEM | 0.78 [0.27; 2.29] | -0.46 | 0.649 | 0.5 | 2 | 0.780 | 0.000 | 0.0 |
|  | CC + CT vs. TT | 3 | FEM | 0.7 [0.26; 1.92] | -0.69 | 0.491 | 1.23 | 2 | 0.539 | 0.000 | 0.0 |
|  | CC vs. CT + TT | 3 | FEM | 1.07 [0.77; 1.48] | 0.39 | 0.694 | 4.3 | 2 | 0.116 | 0.100 | 53.5 |
| Chronic obstructive pulmonary disease (COPD) | C vs. T | 3 | FEM | 0.46 [0.3; 0.71] | -3.54 | 0.000 | 2.95 | 2 | 0.228 | 0.080 | 32.3 |
|  | CC vs. TT | 3 | FEM | 0.59 [0.13; 2.67] | -0.69 | 0.492 | 0.09 | 2 | 0.954 | 0.000 | 0.0 |
|  | CT vs. TT | 3 | FEM | 1.52 [0.31; 7.43] | 0.52 | 0.603 | 0.44 | 2 | 0.804 | 0.000 | 0.0 |
|  | CC + CT vs. TT | 3 | FEM | 0.68 [0.15; 3.09] | -0.5 | 0.620 | 0.1 | 2 | 0.952 | 0.000 | 0.0 |
|  | CC vs. CT + TT | 3 | FEM | 0.43 [0.27; 0.67] | -3.67 | 0.000 | 3.61 | 2 | 0.164 | 0.160 | 44.6 |
| Autoimmune disease | C vs. T | 7 | REM | 1.08 [0.75; 1.56] | 0.42 | 0.672 | 24.95 | 6 | 0.000 | 0.190 | 76.0 |
|  | CC vs. TT | 7 | FEM | 1.02 [0.62; 1.67] | 0.08 | 0.938 | 2.64 | 6 | 0.852 | 0.000 | 0.0 |
|  | CT vs. TT | 7 | FEM | 1.15 [0.68; 1.95] | 0.53 | 0.597 | 1.24 | 6 | 0.975 | 0.000 | 0.0 |
|  | CC + CT vs. TT | 7 | FEM | 1.05 [0.64; 1.72] | 0.2 | 0.842 | 2.18 | 6 | 0.902 | 0.000 | 0.0 |
|  | CC vs. CT + TT | 7 | REM | 1.08 [0.72; 1.64] | 0.38 | 0.703 | 25.99 | 6 | 0.000 | 0.240 | 76.9 |
| Inflammatory disease | C vs. T | 5 | REM | 1.19 [0.8; 1.77] | 0.87 | 0.386 | 12.03 | 4 | 0.017 | 0.130 | 66.7 |
|  | CC vs. TT | 5 | FEM | 1.4 [0.6; 3.28] | 0.78 | 0.437 | 2.28 | 4 | 0.685 | 0.000 | 0.0 |
|  | CT vs. TT | 5 | REM | 0.41 [0.05; 3.06] | -0.87 | 0.383 | 31.58 | 4 | 0.000 | 4.170 | 87.3 |
|  | CC + CT vs. TT | 5 | REM | 0.6 [0.15; 2.34] | -0.74 | 0.458 | 18.68 | 4 | 0.001 | 1.620 | 78.6 |
|  | CC vs. CT + TT | 5 | REM | 2.95 [0.56; 15.54] | 1.28 | 0.201 | 23.83 | 4 | 0.000 | 2.920 | 83.2 |
| Cardiovascular disease (CVD) | C vs. T | 4 | REM | 1.03 [0.71; 1.48] | 0.14 | 0.887 | 13.12 | 3 | 0.004 | 0.090 | 77.1 |
|  | CC vs. TT | 4 | REM | 1.18 [0.46; 3] | 0.34 | 0.733 | 6.71 | 3 | 0.082 | 0.450 | 55.3 |
|  | CT vs. TT | 4 | FEM | 1.09 [0.64; 1.85] | 0.32 | 0.749 | 1.8 | 3 | 0.615 | 0.030 | 0.0 |
|  | CC + CT vs. TT | 4 | FEM | 1.07 [0.64; 1.79] | 0.25 | 0.799 | 4.67 | 3 | 0.198 | 0.280 | 35.7 |
|  | CC vs. CT + TT | 4 | REM | 0.99 [0.66; 1.5] | -0.03 | 0.978 | 11.93 | 3 | 0.008 | 0.110 | 74.9 |
| Skin disase | C vs. T | 2 | REM | 0.83 [0.11; 6.59] | -0.17 | 0.863 | 7.35 | 1 | 0.007 | 1.950 | 86.4 |
|  | CC vs. TT | 2 | FEM | 3.01 [1.09; 8.32] | 2.12 | 0.034 | 0.01 | 1 | 0.905 | 0.000 | 0.0 |
|  | CT vs. TT | 2 | FEM | 1.68 [0.56; 5.11] | 0.92 | 0.357 | 0.58 | 1 | 0.446 | 0.000 | 0.0 |
|  | CC + CT vs. TT | 2 | FEM | 2.71 [0.98; 7.49] | 1.92 | 0.055 | 0 | 1 | 0.997 | 0.000 | 0.0 |
|  | CC vs. CT + TT | 2 | REM | 0.81 [0.09; 6.85] | -0.2 | 0.844 | 7.47 | 1 | 0.006 | 2.090 | 86.6 |
| Type 2 diabetes | C vs. T | 4 | REM | 1.31 [0.85; 2] | 1.23 | 0.218 | 10.34 | 3 | 0.016 | 0.130 | 71.0 |
|  | CC vs. TT | 4 | REM | 2.06 [0.47; 9.08] | 0.95 | 0.340 | 8.23 | 3 | 0.042 | 1.240 | 63.6 |
|  | CT vs. TT | 4 | FEM | 1.65 [0.84; 3.26] | 1.45 | 0.147 | 3.2 | 3 | 0.362 | 0.300 | 6.3 |
|  | CC + CT vs. TT | 4 | REM | 2.05 [0.53; 7.96] | 1.03 | 0.301 | 6.98 | 3 | 0.073 | 0.960 | 57.0 |
|  | CC vs. CT + TT | 4 | REM | 1.33 [0.9; 1.95] | 1.45 | 0.148 | 6.3 | 3 | 0.098 | 0.080 | 52.4 |
| Diabetic complications | C vs. T | 5 | FEM | 1.34 [1.12; 1.61] | 3.22 | 0.001 | 3.26 | 4 | 0.516 | 0.000 | 0.0 |
|  | CC vs. TT | 5 | FEM | 1.59 [0.94; 2.69] | 1.72 | 0.085 | 1.34 | 4 | 0.855 | 0.000 | 0.0 |
|  | CT vs. TT | 5 | FEM | 2.43 [1.41; 4.18] | 3.19 | 0.001 | 3.88 | 4 | 0.422 | 0.160 | 0.0 |
|  | CC + CT vs. TT | 5 | FEM | 2.11 [1.29; 3.43] | 3 | 0.003 | 0.37 | 4 | 0.985 | 0.000 | 0.0 |
|  | CC vs. CT + TT | 5 | REM | 1.24 [0.88; 1.75] | 1.24 | 0.216 | 8.12 | 4 | 0.087 | 0.070 | 50.7 |
| Others | C vs. T | 5 | FEM | 1.1 [0.91; 1.32] | 1.02 | 0.309 | 4.69 | 4 | 0.320 | 0.030 | 14.8 |
|  | CC vs. TT | 5 | FEM | 0.95 [0.59; 1.52] | -0.23 | 0.822 | 3.28 | 4 | 0.513 | 0.000 | 0.0 |
|  | CT vs. TT | 5 | FEM | 0.95 [0.61; 1.5] | -0.2 | 0.841 | 2.88 | 4 | 0.578 | 0.000 | 0.0 |
|  | CC + CT vs. TT | 5 | FEM | 0.96 [0.62; 1.48] | -0.19 | 0.852 | 3.26 | 4 | 0.516 | 0.000 | 0.0 |
|  | CC vs. CT + TT | 5 | FEM | 1.18 [0.93; 1.49] | 1.34 | 0.179 | 4.11 | 4 | 0.391 | 0.030 | 2.8 |
| Asian | C vs. T | 22 | REM | 0.97 [0.81; 1.17] | -0.27 | 0.785 | 56.5 | 21 | 0.000 | 0.100 | 62.8 |
|  | CC vs. TT | 22 | FEM | 0.87 [0.66; 1.14] | -1.03 | 0.305 | 9.76 | 21 | 0.982 | 0.000 | 0.0 |
|  | CT vs. TT | 22 | FEM | 1.19 [0.99; 1.45] | 1.83 | 0.067 | 17.06 | 21 | 0.708 | 0.050 | 0.0 |
|  | CC + CT vs. TT | 22 | FEM | 1.1 [0.92; 1.32] | 1.04 | 0.300 | 14.88 | 21 | 0.829 | 0.020 | 0.0 |
|  | CC vs. CT + TT | 22 | REM | 0.92 [0.73; 1.15] | -0.75 | 0.455 | 56.27 | 21 | 0.000 | 0.160 | 62.7 |
| Caucasian | C vs. T | 11 | REM | 1.27 [1.05; 1.54] | 2.49 | 0.013 | 29.61 | 10 | 0.001 | 0.070 | 66.2 |
|  | CC vs. TT | 11 | FEM | 2 [1.4; 2.87] | 3.78 | 0.000 | 7.72 | 10 | 0.656 | 0.000 | 0.0 |
|  | CT vs. TT | 11 | FEM | 1.64 [1.12; 2.4] | 2.55 | 0.011 | 2.73 | 10 | 0.987 | 0.000 | 0.0 |
|  | CC + CT vs. TT | 11 | FEM | 1.93 [1.35; 2.77] | 3.6 | 0.000 | 6.43 | 10 | 0.778 | 0.000 | 0.0 |
|  | CC vs. CT + TT | 11 | REM | 1.24 [1.02; 1.52] | 2.15 | 0.032 | 26.02 | 10 | 0.004 | 0.060 | 61.6 |
| Mixed | C vs. T | 5 | REM | 1.61 [0.83; 3.12] | 1.4 | 0.160 | 19 | 4 | 0.001 | 0.430 | 79.0 |
|  | CC vs. TT | 5 | FEM | 0.92 [0.37; 2.3] | -0.17 | 0.866 | 3.34 | 4 | 0.502 | 0.430 | 0.0 |
|  | CT vs. TT | 5 | REM | 0.18 [0.03; 1.31] | -1.69 | 0.091 | 15.27 | 4 | 0.004 | 3.000 | 73.8 |
|  | CC + CT vs. TT | 5 | FEM | 0.24 [0.11; 0.54] | -3.48 | 0.001 | 7.16 | 4 | 0.128 | 1.100 | 44.2 |
|  | CC vs. CT + TT | 5 | REM | 3.38 [0.79; 14.41] | 1.65 | 0.100 | 29.58 | 4 | 0.000 | 2.320 | 86.5 |
| **rs11549467 (1790 G/A)** | | | | | | | | | | | |
| Overall | A vs. G | 24 | REM | 1.03 [0.81; 1.31] | 0.26 | 0.795 | 75.04 | 23 | 0.000 | 0.18 | 69.4 |
|  | AA vs. GG | 24 | FEM | 0.96 [0.75; 1.23] | -0.31 | 0.753 | 32.43 | 9 | 0.000 | 0.42 | 72.3 |
|  | AG vs. GG | 24 | REM | 1.18 [0.8; 1.72] | 0.84 | 0.402 | 99.55 | 23 | 0.000 | 0.57 | 76.9 |
|  | AA + AG vs. GG | 24 | REM | 1.1 [0.79; 1.53] | 0.57 | 0.572 | 89.84 | 23 | 0.000 | 0.41 | 74.4 |
|  | AA vs. AG + GG | 24 | FEM | 0.78 [0.67; 0.91] | -3.1 | 0.002 | 6.5 | 23 | 1.000 | 0 | 0.0 |
| Preeclampsia | A vs. G | 2 | FEM | 0.63 [0.27; 1.43] | -1.11 | 0.269 | 0.4 | 1 | 0.525 | 0 | 0.0 |
|  | AA vs. GG | 2 | REM | NA [NA; NA] | NA | NA | NA | 1 | 0.000 | NA | NA |
|  | AG vs. GG | 2 | FEM | 0.62 [0.27; 1.43] | -1.12 | 0.263 | 0.4 | 1 | 0.529 | 0 | 0.0 |
|  | AA + AG vs. GG | 2 | FEM | 0.62 [0.27; 1.43] | -1.12 | 0.263 | 0.4 | 1 | 0.529 | 0 | 0.0 |
|  | AA vs. AG + GG | 2 | FEM | 0.84 [0.05; 13.43] | -0.13 | 0.900 | 0.02 | 1 | 0.902 | 0 | 0.0 |
| Inflammatory disease | A vs. G | 5 | FEM | 0.86 [0.7; 1.06] | -1.38 | 0.166 | 3.25 | 4 | 0.517 | 0 | 0.0 |
|  | AA vs. GG | 5 | FEM | 0.99 [0.21; 4.63] | -0.01 | 0.992 | 0.53 | 4 | 0.971 | 0 | 0.0 |
|  | AG vs. GG | 5 | FEM | 0.98 [0.65; 1.46] | -0.12 | 0.904 | 4.32 | 4 | 0.364 | 0.21 | 7.5 |
|  | AA + AG vs. GG | 5 | FEM | 0.98 [0.66; 1.44] | -0.12 | 0.904 | 3.29 | 4 | 0.511 | 0.06 | 0.0 |
|  | AA vs. AG + GG | 5 | FEM | 0.81 [0.62; 1.05] | -1.62 | 0.106 | 0.38 | 4 | 0.984 | 0 | 0.0 |
| Chronic obstructive pulmonary disease (COPD) | A vs. G | 3 | REM | 1.54 [0.32; 7.34] | 0.54 | 0.588 | 16.77 | 2 | 0.000 | 1.67 | 88.1 |
|  | AA vs. GG | 3 | FEM | 2.61 [0.51; 13.29] | 1.15 | 0.249 | 0 | 2 | 1.000 | NA | NA |
|  | AG vs. GG | 3 | REM | 1.73 [0.29; 10.55] | 0.6 | 0.550 | 19.78 | 2 | 0.000 | 2.28 | 89.9 |
|  | AA + AG vs. GG | 3 | REM | 1.67 [0.3; 9.41] | 0.58 | 0.562 | 19.11 | 2 | 0.000 | 2.08 | 89.5 |
|  | AA vs. AG + GG | 3 | FEM | 1.82 [0.43; 7.79] | 0.81 | 0.417 | 0.13 | 2 | 0.935 | 0 | 0.0 |
| Autoimmune disease | A vs. G | 2 | FEM | 0.9 [0.47; 1.72] | -0.33 | 0.742 | 0.54 | 1 | 0.463 | 0 | 0.0 |
|  | AA vs. GG | 2 | FEM | 0.13 [0; 6.57] | -1.02 | 0.308 | 0 | 1 | 1.000 | NA | NA |
|  | AG vs. GG | 2 | FEM | 1 [0.5; 1.98] | -0.01 | 0.996 | 0.65 | 1 | 0.420 | 0 | 0.0 |
|  | AA + AG vs. GG | 2 | FEM | 0.94 [0.48; 1.86] | -0.16 | 0.869 | 0.59 | 1 | 0.441 | 0 | 0.0 |
|  | AA vs. AG + GG | 2 | FEM | 0.45 [0.04; 5.09] | -0.64 | 0.522 | 0.14 | 1 | 0.705 | 0 | 0.0 |
| Cardiovascular disease (CVD) | A vs. G | 4 | FEM | 0.83 [0.67; 1.02] | -1.75 | 0.080 | 6.12 | 3 | 0.106 | 0.07 | 51.0 |
|  | AA vs. GG | 4 | FEM | 0.46 [0.25; 0.84] | -2.51 | 0.012 | 0 | 3 | 1.000 | NA | NA |
|  | AG vs. GG | 4 | REM | 0.82 [0.5; 1.35] | -0.77 | 0.441 | 6.63 | 3 | 0.085 | 0.13 | 54.7 |
|  | AA + AG vs. GG | 4 | REM | 0.79 [0.47; 1.34] | -0.87 | 0.385 | 7.85 | 3 | 0.049 | 0.16 | 61.8 |
|  | AA vs. AG + GG | 4 | FEM | 0.73 [0.52; 1.03] | -1.78 | 0.076 | 0.08 | 3 | 0.995 | 0 | 0.0 |
| Type 2 diabetes | A vs. G | 3 | FEM | 1.26 [0.99; 1.6] | 1.87 | 0.062 | 0.78 | 2 | 0.678 | 0 | 0.0 |
|  | AA vs. GG | 3 | FEM | 1.48 [0.89; 2.46] | 1.52 | 0.128 | 0.49 | 2 | 0.783 | 0 | 0.0 |
|  | AG vs. GG | 3 | REM | 1.86 [0.71; 4.85] | 1.26 | 0.207 | 14.98 | 2 | 0.001 | 0.59 | 86.7 |
|  | AA + AG vs. GG | 3 | REM | 1.54 [0.85; 2.79] | 1.42 | 0.155 | 6.73 | 2 | 0.035 | 0.18 | 70.3 |
|  | AA vs. AG + GG | 3 | FEM | 0.79 [0.5; 1.23] | -1.04 | 0.299 | 0.04 | 2 | 0.983 | 0 | 0.0 |
| Diabetic complications | A vs. G | 2 | FEM | 1.71 [1.27; 2.28] | 3.58 | 0.000 | 0.4 | 1 | 0.525 | 0 | 0.0 |
|  | AA vs. GG | 2 | FEM | 2.34 [1.4; 3.89] | 3.27 | 0.001 | 0 | 1 | 1.000 | NA | NA |
|  | AG vs. GG | 2 | REM | 2.83 [0.65; 12.31] | 1.39 | 0.166 | 6.27 | 1 | 0.012 | 0.95 | 84.0 |
|  | AA + AG vs. GG | 2 | REM | 2.3 [0.89; 5.96] | 1.71 | 0.087 | 3.03 | 1 | 0.082 | 0.33 | 67.0 |
|  | AA vs. AG + GG | 2 | FEM | 1.07 [0.69; 1.66] | 0.31 | 0.759 | 0 | 1 | 0.959 | 0 | 0.0 |
| Others | A vs. G | 3 | FEM | 0.72 [0.58; 0.89] | -2.97 | 0.003 | 2.69 | 2 | 0.260 | 0 | 25.7 |
|  | AA vs. GG | 3 | FEM | 0.5 [0.32; 0.78] | -3.07 | 0.002 | 0.4 | 2 | 0.819 | 0 | 0.0 |
|  | AG vs. GG | 3 | FEM | 0.82 [0.58; 1.15] | -1.16 | 0.246 | 2.37 | 2 | 0.307 | 0 | 15.4 |
|  | AA + AG vs. GG | 3 | FEM | 0.72 [0.52; 0.99] | -2.04 | 0.042 | 2.97 | 2 | 0.226 | 0 | 32.8 |
|  | AA vs. AG + GG | 3 | FEM | 0.6 [0.41; 0.87] | -2.67 | 0.008 | 0.16 | 2 | 0.922 | 0 | 0.0 |
| Asian | A vs. G | 15 | REM | 1.13 [0.84; 1.52] | 0.79 | 0.430 | 66.5 | 14 | 0.000 | 0.23 | 79.0 |
|  | AA vs. GG | 15 | REM | 0.94 [0.49; 1.8] | -0.19 | 0.850 | 31.91 | 14 | 0.004 | 0.49 | 78.1 |
|  | AG vs. GG | 15 | REM | 1.47 [0.92; 2.37] | 1.59 | 0.111 | 84.33 | 14 | 0.000 | 0.66 | 83.4 |
|  | AA + AG vs. GG | 15 | REM | 1.3 [0.86; 1.99] | 1.24 | 0.217 | 77.34 | 14 | 0.000 | 0.49 | 81.9 |
|  | AA vs. AG + GG | 15 | FEM | 0.78 [0.67; 0.91] | -3.11 | 0.002 | 6.01 | 14 | 0.966 | 0 | 0.0 |
| Caucasian | AA vs. GG | 4 | FEM | 4.27 [0.06; 294.61] | 0.67 | 0.502 | 0 | 3 | 1.000 | NA | NA |
|  | A vs. G | 4 | FEM | 0.94 [0.63; 1.39] | -0.32 | 0.749 | 6.04 | 3 | 0.110 | 0.3 | 50.3 |
|  | AG vs. GG | 4 | REM | 0.57 [0.21; 1.54] | -1.11 | 0.268 | 7.81 | 3 | 0.050 | 0.55 | 61.6 |
|  | AA + AG vs. GG | 4 | REM | 0.63 [0.26; 1.52] | -1.02 | 0.308 | 6.85 | 3 | 0.077 | 0.39 | 56.2 |
|  | AA vs. AG + GG | 4 | FEM | 1.17 [0.19; 7.34] | 0.17 | 0.867 | 0.28 | 3 | 0.963 | 0 | 0.0 |
| Mixed | A vs. G | 5 | FEM | 0.96 [0.68; 1.34] | -0.26 | 0.795 | 2.45 | 4 | 0.653 | 0 | 0.0 |
|  | AA vs. GG | 5 | FEM | 0.79 [0.15; 4.15] | -0.27 | 0.785 | 0 | 4 | 1.000 | NA | NA |
|  | AG vs. GG | 5 | FEM | 0.97 [0.67; 1.41] | -0.16 | 0.869 | 2.64 | 4 | 0.621 | 0 | 0.0 |
|  | AA + AG vs. GG | 5 | FEM | 0.96 [0.67; 1.39] | -0.21 | 0.831 | 2.57 | 4 | 0.633 | 0 | 0.0 |
|  | AA vs. AG + GG | 5 | FEM | 0.81 [0.22; 2.93] | -0.32 | 0.747 | 0.03 | 4 | 1.000 | 0 | 0.0 |
