# Supplementary material for: Association of hypoxia inducible factor 1-Alpha gene polymorphisms with multiple disease risks: A comprehensive meta-analysis
Source: PLoS One. 2022 Aug 16;17(8):e0273042. doi: 10.1371/journal.pone.0273042 (PMC9380912; doi:10.1371/journal.pone.0273042)
Supplement: S3 Table — (DOCX) [file pone.0273042.s007.docx]

**Table S2.** Publication bias using the Begg’s and Egger’s test of the HIF1A 1722 T/C and 1790 G/A polymorphisms

| **Polymorphisms** | **Begg’s Test** | | | **Egger’s Test** | | | |
| --- | --- | --- | --- | --- | --- | --- | --- |
|  | **z-value** | ***P*** | **Comment** | **t-value** | **d.f** | ***P*** | **Comment** |
| **HIF1A 1772 T/C** | | | | | | | |
| CC vs. TT | 0.34 | 0.7343 | no publication bias | 1.30 | 36 | 0.2020 | no publication bias |
| C vs. T | -0.01 | 0.9900 | no publication bias | 0.67 | 36 | 0.5052 | no publication bias |
| **HIF1A 1790 G/A** | | | | | | | |
| A vs. G | -0.35 | 0.7284 | no publication bias | 0.19 | 22 | 0.8537 | no publication bias |
| **Hypothesis testing by *p*-value for checking publication bias** | | | | | | | |
| H_0_: Null Hypothesis (Symmetry in the funnel Plot) | | | | | | | |
| H_1_: Alternative Hypothesis (Asymmetry in the funnel Plot) | | | | | | | |
| For *P* ≤ 0.05 we Reject Null Hypothesis (H_0_) | | | | | | | |
| For *P* > 0.05 we Accept Null Hypothesis (H_0_) and Reject Alternative Hypothesis (H_1_) | | | | | | | |
