# Supplementary material for: Association of hypoxia inducible factor 1-Alpha gene polymorphisms with multiple disease risks: A comprehensive meta-analysis
Source: PLoS One. 2022 Aug 16;17(8):e0273042. doi: 10.1371/journal.pone.0273042 (PMC9380912; doi:10.1371/journal.pone.0273042)
Supplement: S4 Table — (DOCX) [file pone.0273042.s008.docx]

**Table S3.** Group of disease conducting this meta-analysis

| **Disease group name** | **rs11549465** | **rs11549467** |
| --- | --- | --- |
| Autoimmune disease | Primary Sjogrens syndrome,  Systemic lupus erythematosus,  Type 1 diabetes,  Systemic sclerosis,  Psoriasisÿ ,  Multiple sclerosis | Primary Sjogrens syndrome,  Systemic sclerosis |
| Cardiovascular disease (CVD) | Coronary artery disease (CAD),  Left ventricular hypertrophy | Coronary artery disease (CAD),  Peripheral artery disease (PAD),  Left ventricular hypertrophy |
| Chronic obstructive pulmonary disease (COPD) | Chronic obstructive pulmonary disease (COPD) | Chronic obstructive pulmonary disease (COPD) |
| Diabetic complications | Diabetic retinopathy,  Diabetic food ulcer,  Diabetic nephropathy | Diabetic food ulcer,  Diabetic nephropathy |
| Inflammatory disease | Osteoarthritis,  Giant cell arteritis,  Oral lichen planus (OLP),  Parkinson's disease | Inflammatory bowel disease (IBD),  Osteoarthritis,  Giant cell arteritis,  Osteoarthritis,  Parkinson's disease |
| Preeclampsia | Preeclampsia | Preeclampsia |
| Skin disease | Cellulitis,  Pressure injury | - |
| Type 2 diabetes | Type 2 diabetes | Type 2 diabetes |
| Others | Age-related macular degeneration (AMD),  Hemodialysis,  Lumbar disc degeneration (LDD),  High altitude polycythemia (HAPC),  Metabolic syndrome, | Lumbar disc degeneration (LDD),  High altitude polycythemia (HAPC),  Pressure injury |
