# Supplementary material for: Association of hypoxia inducible factor 1-Alpha gene polymorphisms with multiple disease risks: A comprehensive meta-analysis
Source: PLoS One. 2022 Aug 16;17(8):e0273042. doi: 10.1371/journal.pone.0273042 (PMC9380912; doi:10.1371/journal.pone.0273042)
Supplement: S1 File — (DOCX) [file pone.0273042.s009.docx]

**Forest plots**

**
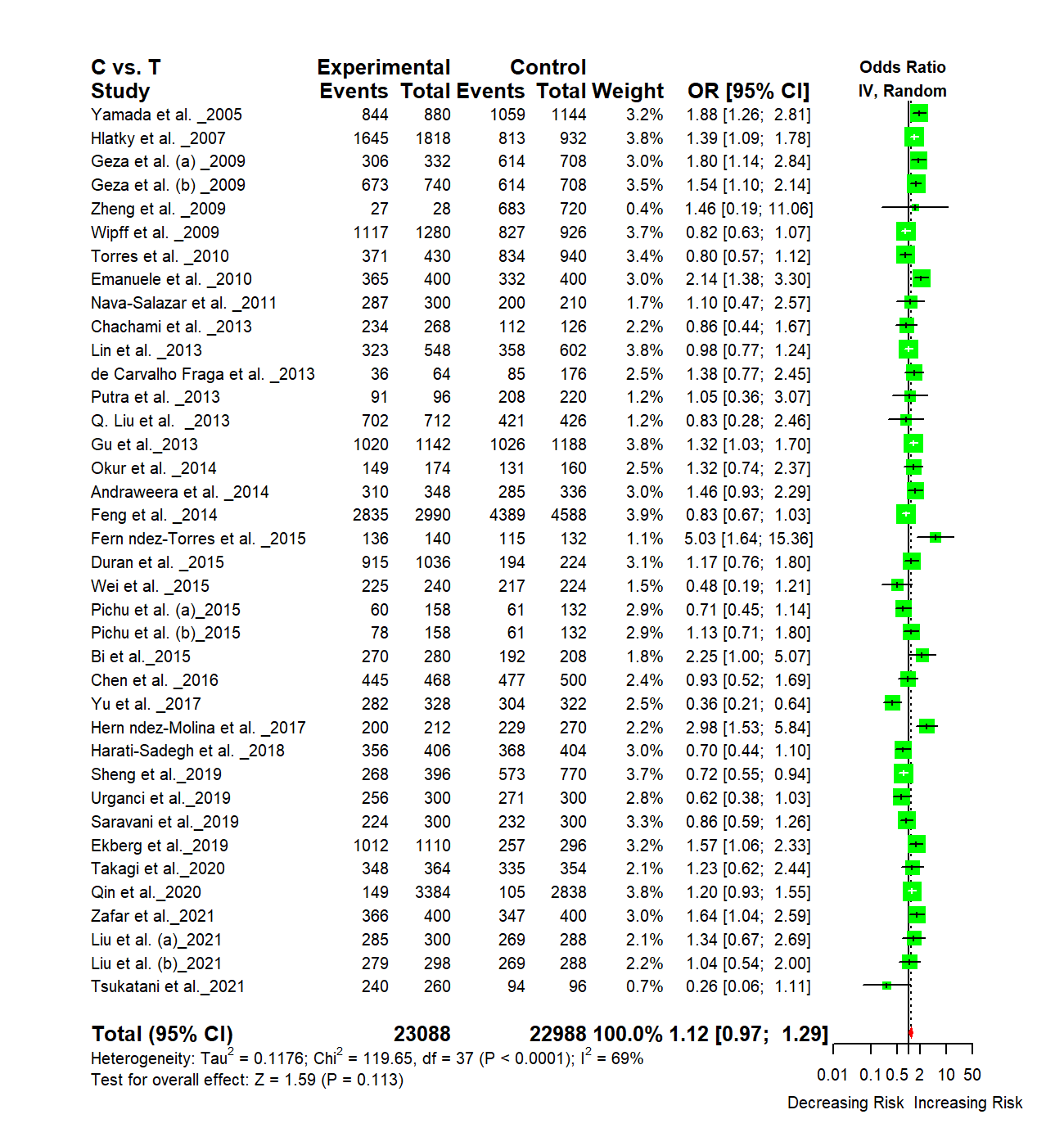
**

**Fig S1.** **Forest plot of HIF1A polymorphism 1772 C/T for overall disease risk under allelic contrast models [*C* vs. *T*].**

**
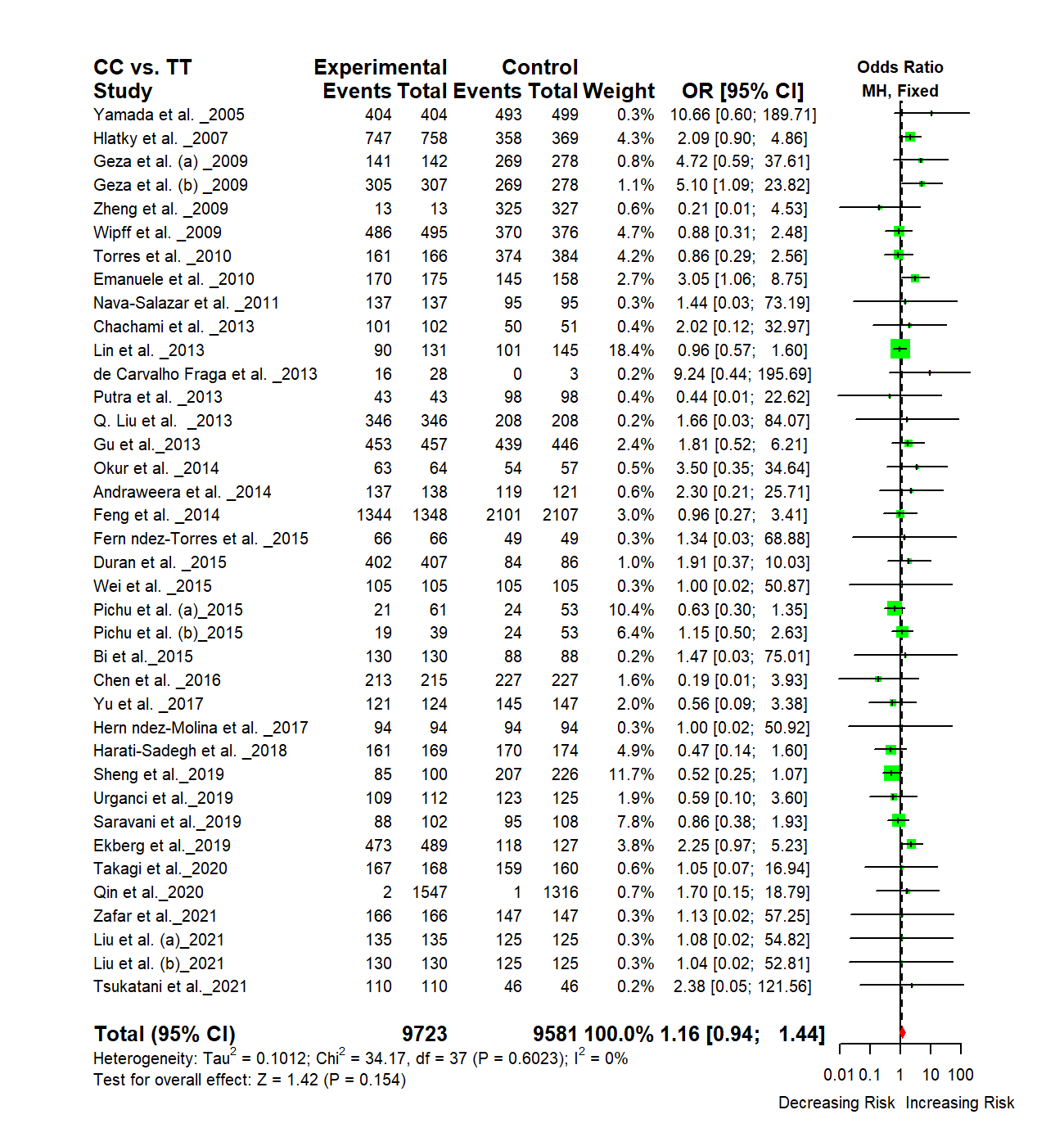
**

**Fig S2.** **Forest plot of HIF1A polymorphism 1772 C/T for overall disease risk under allelic contrast models [*CC* vs. *TT*].**

**
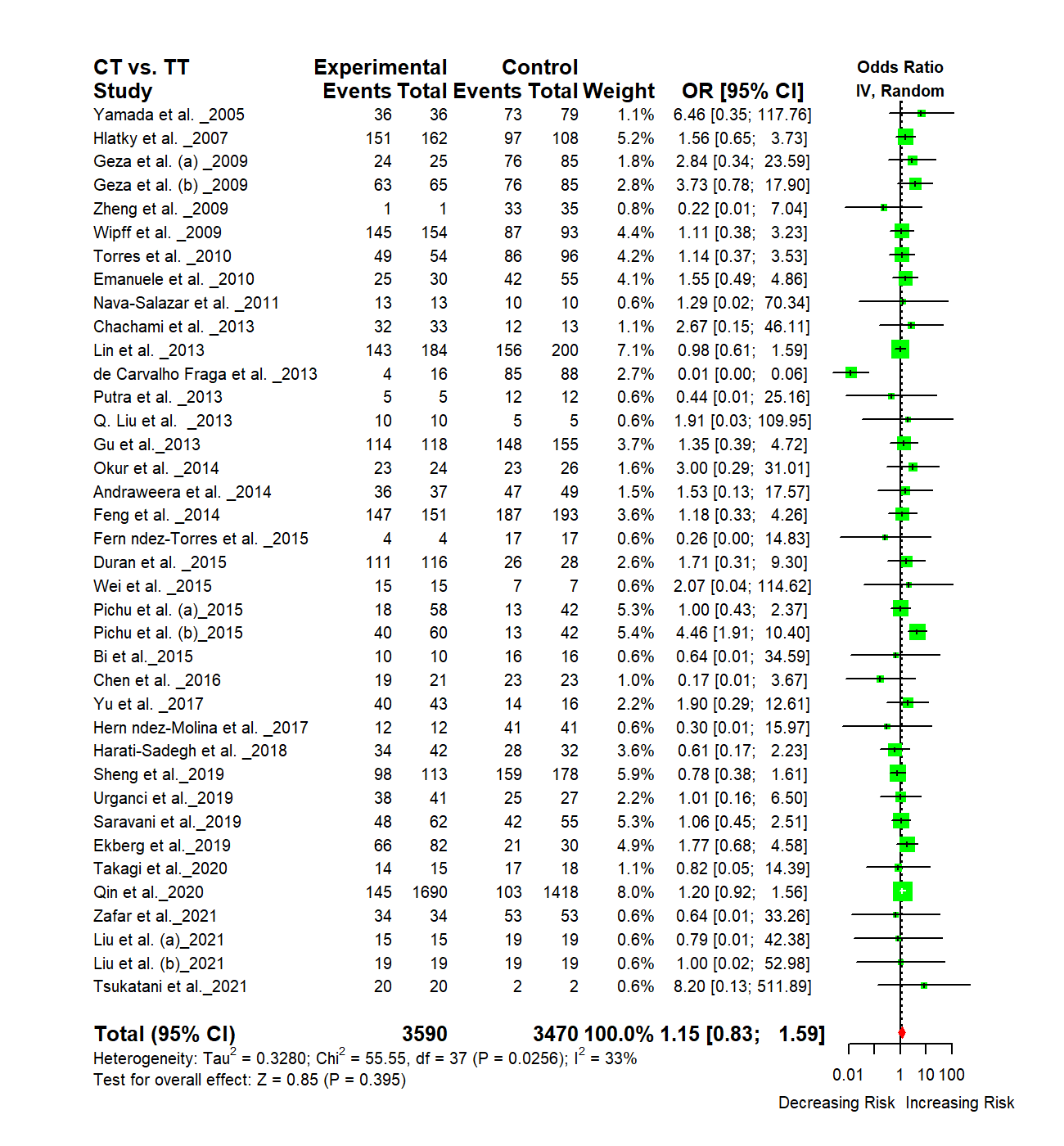
**

**Fig S3.** **Forest plot of HIF1A polymorphism 1772 C/T for overall disease risk under heterozygote models [*CT* vs. *TT*].**

**
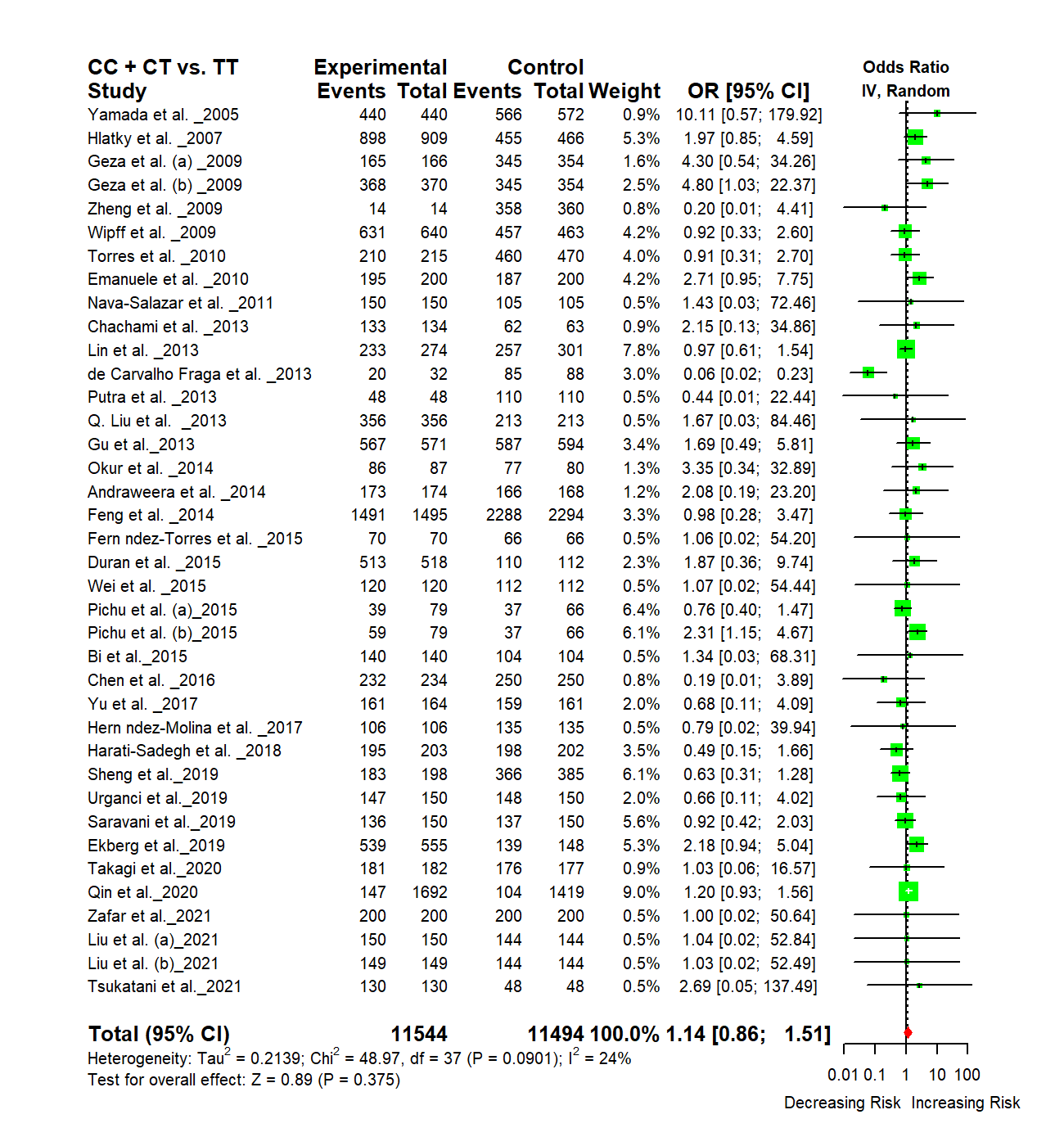
**

**Fig S4.** **Forest plot of HIF1A polymorphism 1772 C/T for overall disease risk under dominant models [*CC* + *CT* vs. *TT*].**

**
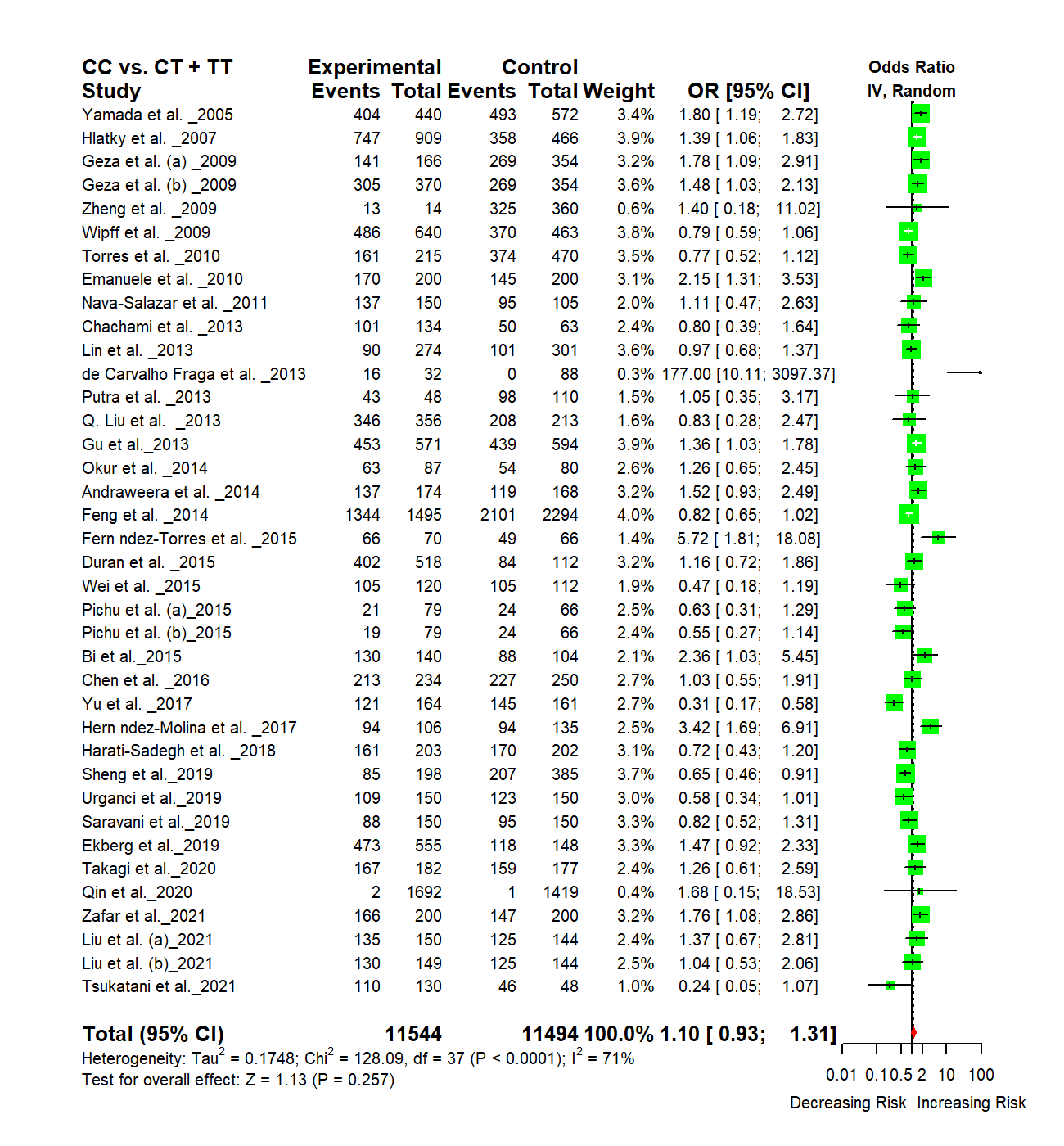
**

**Fig S5.** **Forest plot of HIF1A polymorphism 1772 C/T for overall disease risk under recessive models [*CC* vs. *CT* + *TT*].**


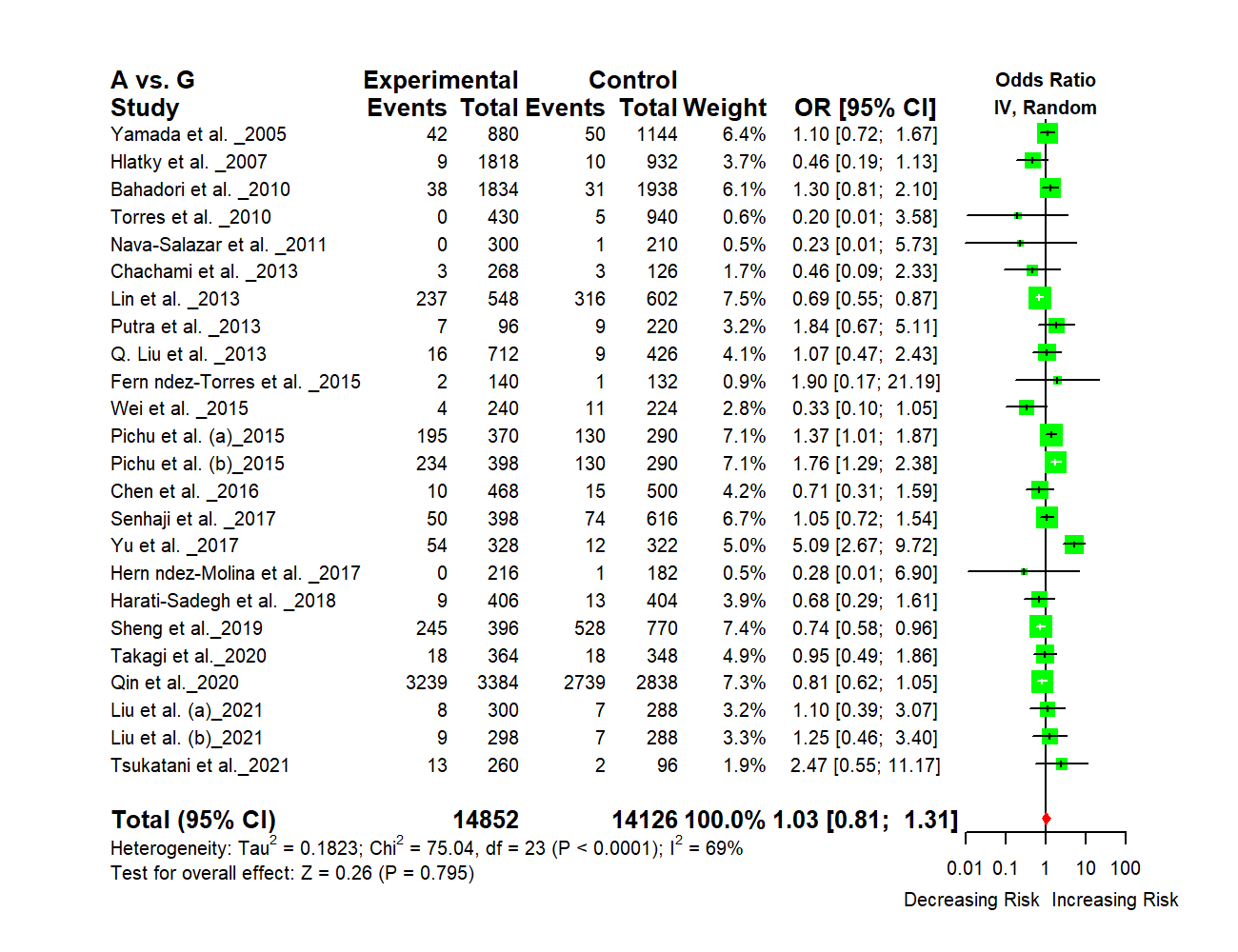


**Fig S6.** **Forest plot of HIF1A polymorphism 1790 G/A for overall disorder risk under allelic contrast models [*A* vs. *G*].**


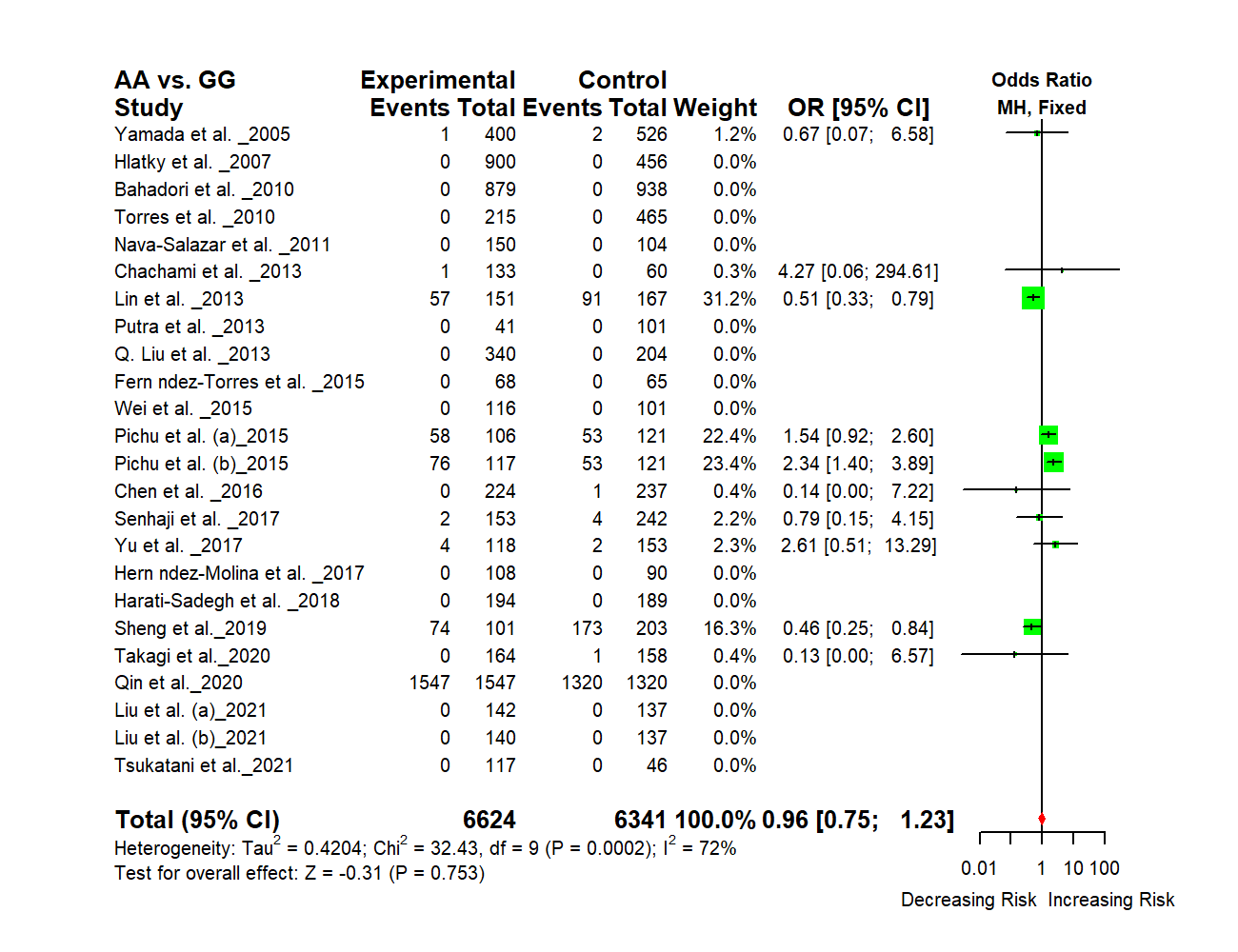


**Fig S7.** **Forest plot of HIF1A polymorphism 1790 G/A for overall disorder risk under allelic contrast models [*AA* vs. *GG*].**


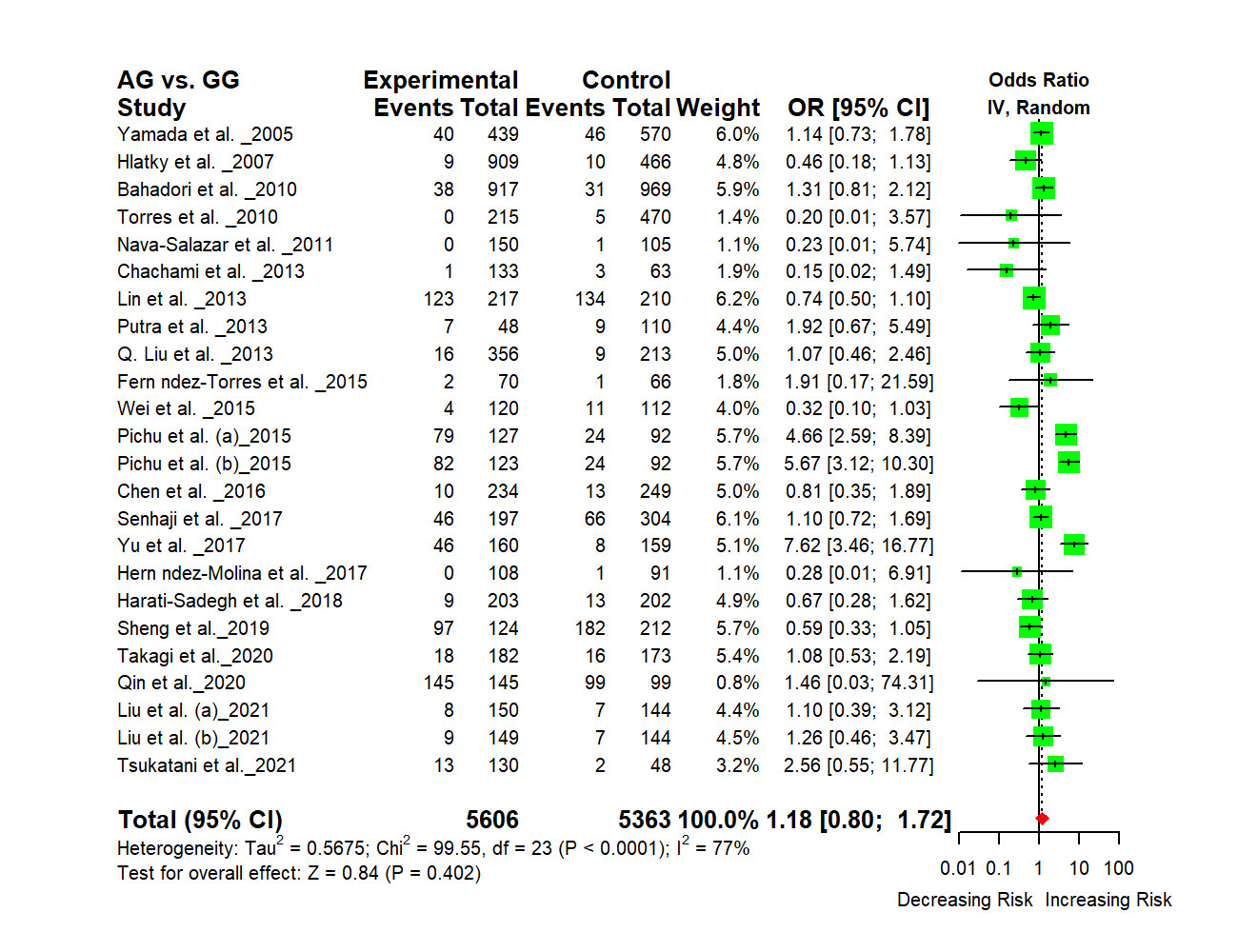


**Fig S8.** **Forest plot of HIF1A polymorphism 1790 G/A for overall disorder risk under heterozygote models [*AG* vs. *GG*].**


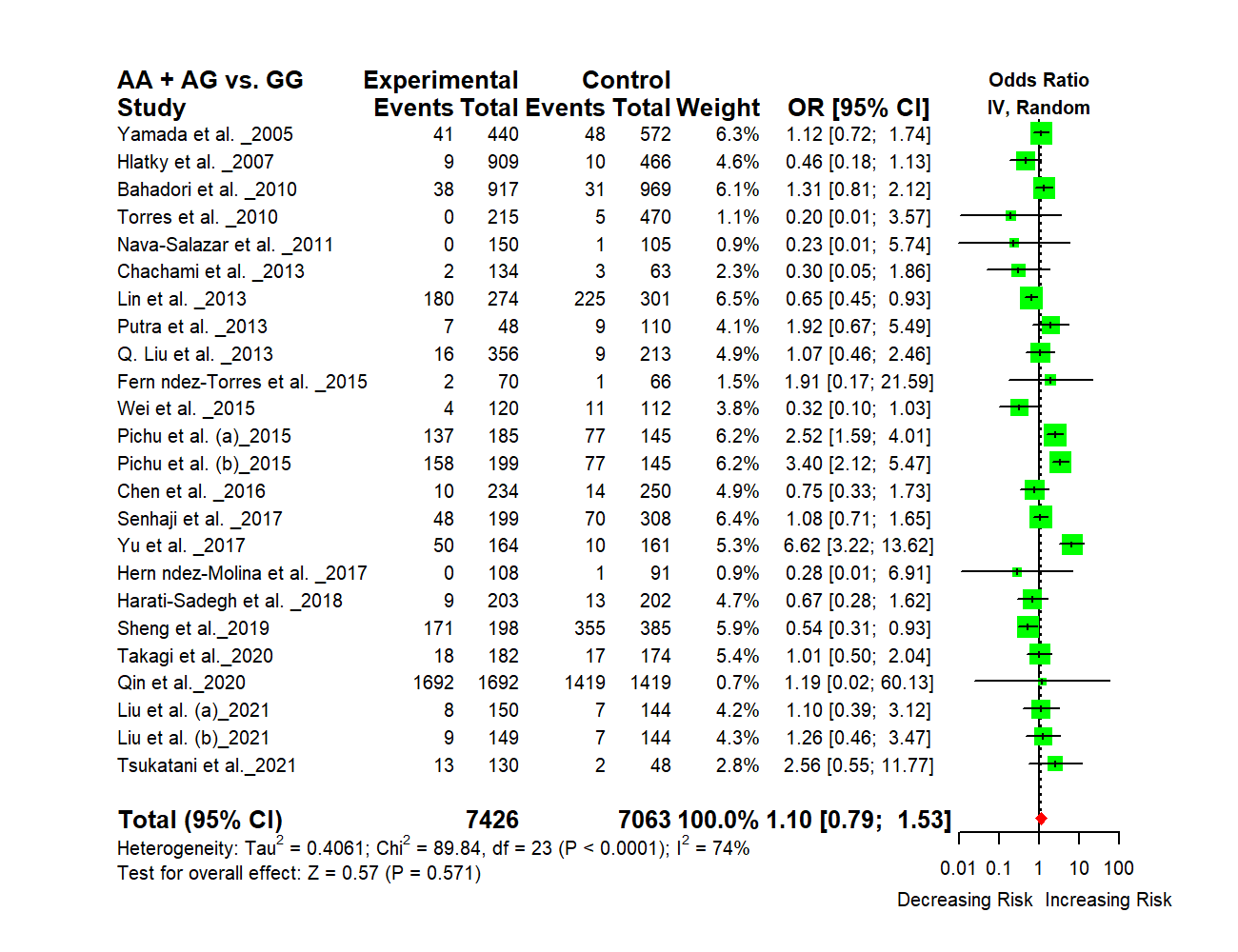


**Fig S9.** **Forest plot of HIF1A polymorphism 1790 G/A for overall disorder risk under dominant models [*AA* + *AG* vs. *GG*].**


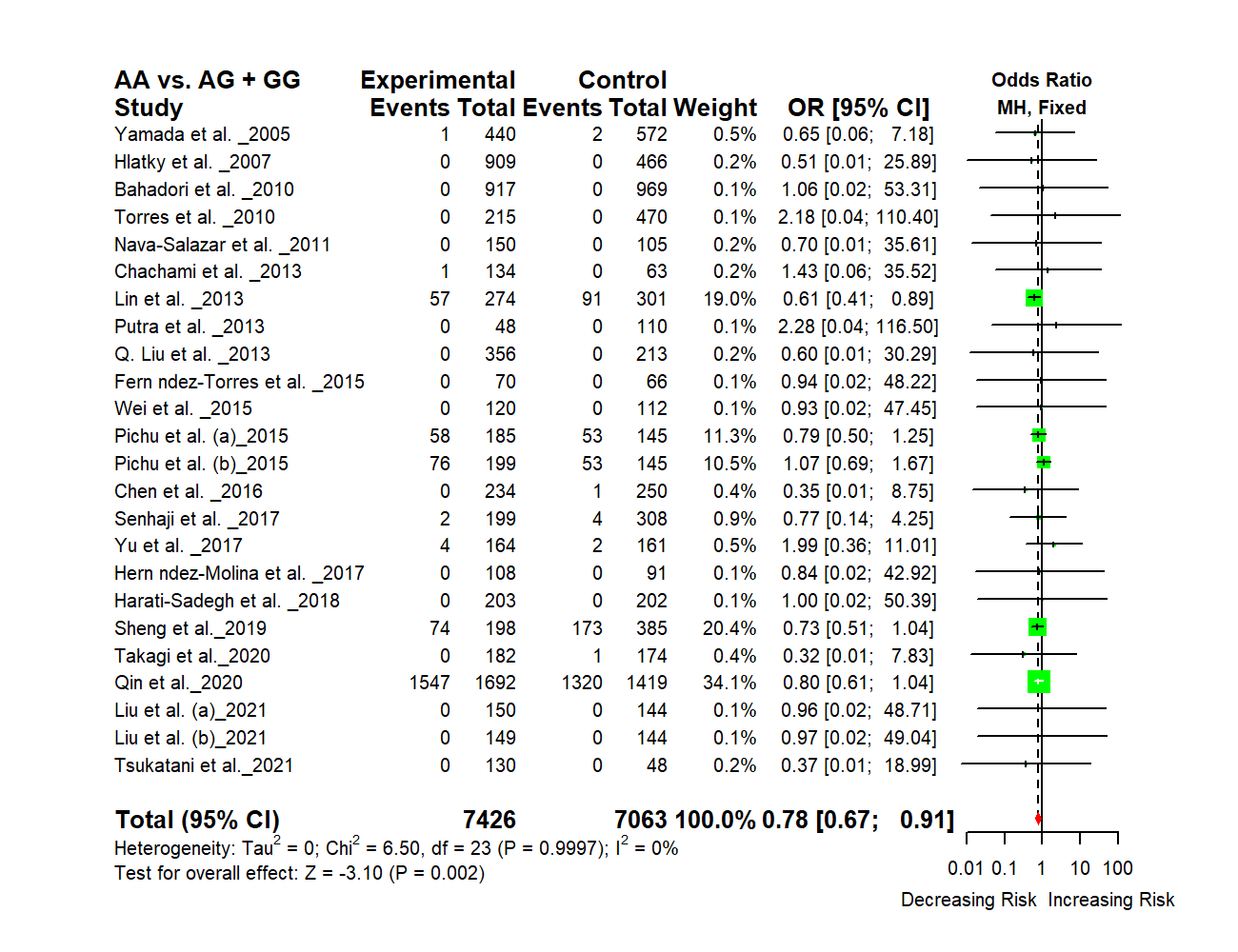


**Fig S10.** **Forest plot of HIF1A polymorphism 1790 G/A for overall disorder risk under recessive models [*AA* vs. *AG* + *GG*].**
